# Efficient prediction of a spatial transcriptomics profile better characterizes breast cancer tissue sections without costly experimentation

**DOI:** 10.1038/s41598-022-07685-4

**Published:** 2022-03-08

**Authors:** Taku Monjo, Masaru Koido, Satoi Nagasawa, Yutaka Suzuki, Yoichiro Kamatani

**Affiliations:** 1 Department of Computational Biology and Medical Sciences, Graduate School of Frontier Sciences, The University of Tokyo, 5-1-5, Kashiwanoha, Kashiwa-shi, Chiba 277-8562 Japan; 2 Division of Molecular Pathology, Department of Cancer Biology, Institute of Medical Science, The University of Tokyo, 4-6-1, Shirokanedai, Minato-ku, Tokyo, 108-8639 Japan; 3 Division of Breast and Endocrine Surgery, Department of Surgery, St. Marianna University School of Medicine, 2-16-1, Sugao, Miyamae-ku, Kawasaki-shi, Kanagawa 216-8511 Japan

**Keywords:** Transcriptomics, Computational biology and bioinformatics, Breast cancer

## Abstract

Spatial transcriptomics is an emerging technology requiring costly reagents and considerable skills, limiting the identification of transcriptional markers related to histology. Here, we show that predicted spatial gene-expression in unmeasured regions and tissues can enhance biologists’ histological interpretations. We developed the Deep learning model for Spatial gene Clusters and Expression, DeepSpaCE, and confirmed its performance using the spatial-transcriptome profiles and immunohistochemistry images of consecutive human breast cancer tissue sections. For example, the predicted expression patterns of *SPARC*, an invasion marker, highlighted a small tumor-invasion region difficult to identify using raw spatial transcriptome data alone because of a lack of measurements. We further developed semi-supervised DeepSpaCE using unlabeled histology images and increased the imputation accuracy of consecutive sections, enhancing applicability for a small sample size. Our method enables users to derive hidden histological characters via spatial transcriptome and gene annotations, leading to accelerated biological discoveries without additional experiments.

## Introduction

Spatial transcriptomics with in situ capturing is an emerging technology that maps gene-expression profiles with corresponding spatial information in a tissue section^1–4^. A highly resolved spatial-transcriptome profile is invaluable for revealing biological functions and molecular mechanisms^5^. Recently, many histological transcriptome profiles, measured by in situ capturing platforms (numerous spots with barcoded oligonucleotides on a chip), were reported in the field of oncology^6,7^. These profiles have helped demonstrate the complexity and heterogeneity of cancer tissues. For example, histological transcriptome profiles were used to identify high-risk invasive populations in ductal carcinoma tissues using an in situ capturing method^8,9^. However, the experimental cost of spatial transcriptomics, such as for designed chips, reagents, and sequencing, is currently high. It is also challenging to balance spatial resolution (i.e., the density of spots in a tissue slide) and RNA-detection efficiency with current spatial-transcriptome technology^10^. In addition, this technique requires practiced skills to obtain high-quality expression profiles for entire tissue slides, even when using a commercial kit such as the 10x Genomics Visium platform.

The convolutional neural network (CNN), a deep-learning method, is frequently used to discover features from imaging datasets and can predict image categories of interest in an end-to-end manner. For example, in the biomedical field, the CNN method has successfully been used to classify lung cancer subtypes from tissue-section images without prior knowledge^11^. Based on these recent advances, we hypothesized that applying the CNN method to spatial-transcriptome profiles would enable expression-level predictions from hematoxylin and eosin (H&E)-stained section images, potentially leading to an increased number of pixels by predicting spatial gene-expression gaps among spots measured by spatial-transcriptome techniques (super-resolution which was inspired by the recent super-resolution technique^12^) or imputing spatial-transcriptomic patterns which are missing among the consecutive sections (tissue section imputation).

Here, we developed the Deep learning model for Spatial gene Clusters and Expression (DeepSpaCE), which predicts spatial-transcriptome profiles from H&E-stained images using CNNs. First, we verified the accuracy of DeepSpaCE with independent training and testing datasets in a tissue section. We split spot-level expression levels into two sets with corresponding image data. One is for training a prediction model and the other is for validating the accuracy of predicted expression with its real expression profiles. We trained a prediction model using training datasets and verified the prediction accuracy of the model by comparing expression profiles from testing datasets in the fivefold cross-validation. Furthermore, we verified protein-expression patterns in adjacent sections using immunohistochemistry experiments. Based on these verifications, we applied DeepSpaCE for super-resolution of spatial gene-expression levels and imputation of spatial gene-expression levels in other tissue sections using human breast cancer datasets. We did not consider batch effects regarding spatial expressions because we used the Visium dataset developed in the same institute and conditions. Regarding the images, image augmentation was used in the training process to reduce batch effects, if any. It is difficult to obtain many samples in a typical laboratory since Visium experiments are expensive. Therefore, we aimed at maximizing accuracy and utilization with a small sample size.

## Results

### Overview of DeepSpaCE

**Figure 1:**
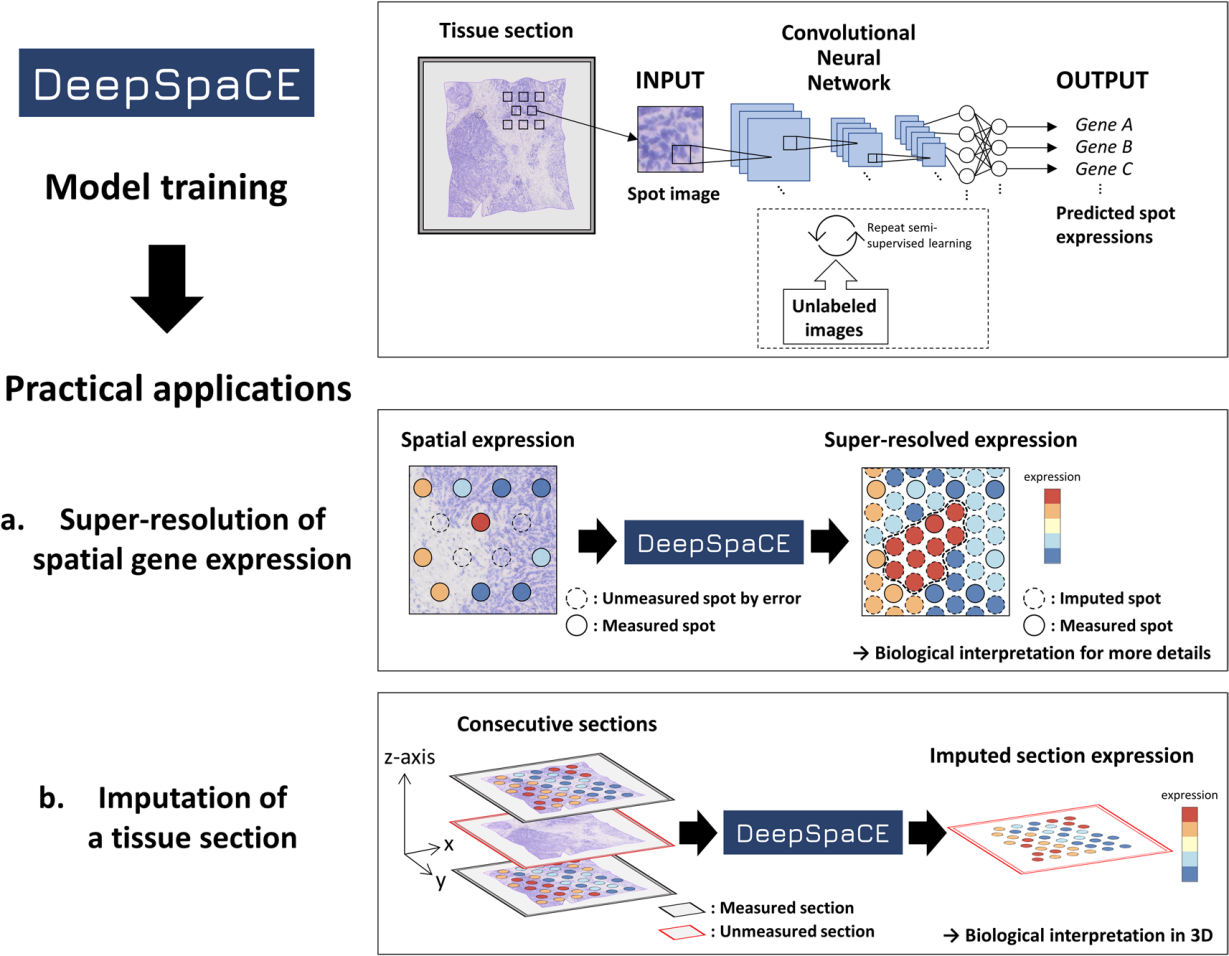
Overview of DeepSpaCE. Deep learning model for Spatial gene Clusters and Expression (DeepSpaCE) is a method for predicting gene-expression levels and transcriptomic cluster types from tissue spot images. DeepSpaCE is composed of two parts: the model training and gene-prediction parts. In the case of using semi-supervised learning as an option, unlabeled images are used to improve the prediction accuracy with predicted proxy labels. As practical applications of DeepSpaCE, we conducted super-resolution of spatial gene expression and tissue section imputation. (**a**) Super-resolution was used for predictions with unmeasured spot images (e.g., images among spots whose expression profiles were measured using the in situ capturing platform or images on spots with technical errors). Left spatial expression pattern shows that some spots lack expression value because of a technical problem such as potential permeabilization error (dotted circle). Right image shows an additional spatial expression pattern imputed by DeepSpaCE, and its highly expressed region in the center of the section (dotted line). It is challenging to infer a functional boundary such as cancer infiltration from spatial expression profiles of sparse spots (left). Spatial expression profiles of dense spots imputed by DeepSpaCE and their gene annotations enable delineating a functional boundary. (**b**) Tissue section imputation was used to predict gene-expression levels in one of the tissue sections within consecutive sections. By using DeepSpaCE, the unmeasured spatial expression profiles of the slide (red frame) can be imputed by at least one adjacent slide (black frame) whose expression profiles were measured using the in situ capturing platform.

DeepSpaCE is composed of two parts: the training and gene-prediction parts (Fig. 1). The CNN (VGG16 architecture^13^) is trained with pairs of cropped tissue section images for each spot (spot image) and its gene-expression profiles. Next, the trained model predicts gene-expression levels for at least one transcript (or transcriptomic cluster type) from spot images. We conducted two types of practical applications of DeepSpaCE using the in situ capturing spatial transcriptome dataset: (a) super-resolution and (b) tissue section imputation.

Super-resolution is used to predict transcript levels at inter-spot space and unmeasured spots in the same image (e.g., images among spots whose expression profiles were measured using the in situ capturing platform or images on spots with potential section-permeabilization errors). Tissue section imputation is performed to predict spatial expression profiles of a section from a series of directly measured consecutive sections. These two applications help reduce experimental costs and elucidate biological functions at higher resolution and in three dimensions. Because substantially fewer data points are available than general deep CNN datasets, we implemented the semi-supervised technique in DeepSpaCE to increase prediction accuracy, as described below. All DeepSpaCE codes for Visium, a standardized, commercially available platform for spatial transcriptome, is available on the GitHub repository (https://github.com/tmonjo/DeepSpaCE).

### Preprocessing of spatial expression data

We preprocessed the spatial expression data from three human breast cancer tissue sections (sections A–C) and their consecutive sections (sections D1–D3) (Supplementary Fig. S1). We excluded spots containing few expressed (or measured) genes to filter out spots with potential permeabilization errors and normalized the spatial expression data to improve the training efficiency by reducing noise. Particularly, the filtering step was critical because spatial expression profiling requires very practiced skills for handling tissue slides and treating reagents homogeneously, and few expressed genes may reveal potential permeabilization errors in the spots. Indeed, in our spatial transcriptome datasets of human breast cancer tissues, the right bottom regions in sections D1 and D3, as well as the right upper region in section D2, showed undetected unique molecular identifiers (UMIs), indicating that potential section-permeabilization errors occurred in these regions (Supplementary Fig. S2a,b). Similarly, such undetected regions were observed in the 10x Genomics Visium demo data of human heart tissue (Supplementary Fig. S2c). The Visium user guide shows such an issue as a partially permeabilized tissue^14^. We used these undetected spots to evaluate the performance of section imputation by DeepSpaCE, as shown below.

### Prediction and experimental validation of spatial gene-expression profiles

**Figure 2:**
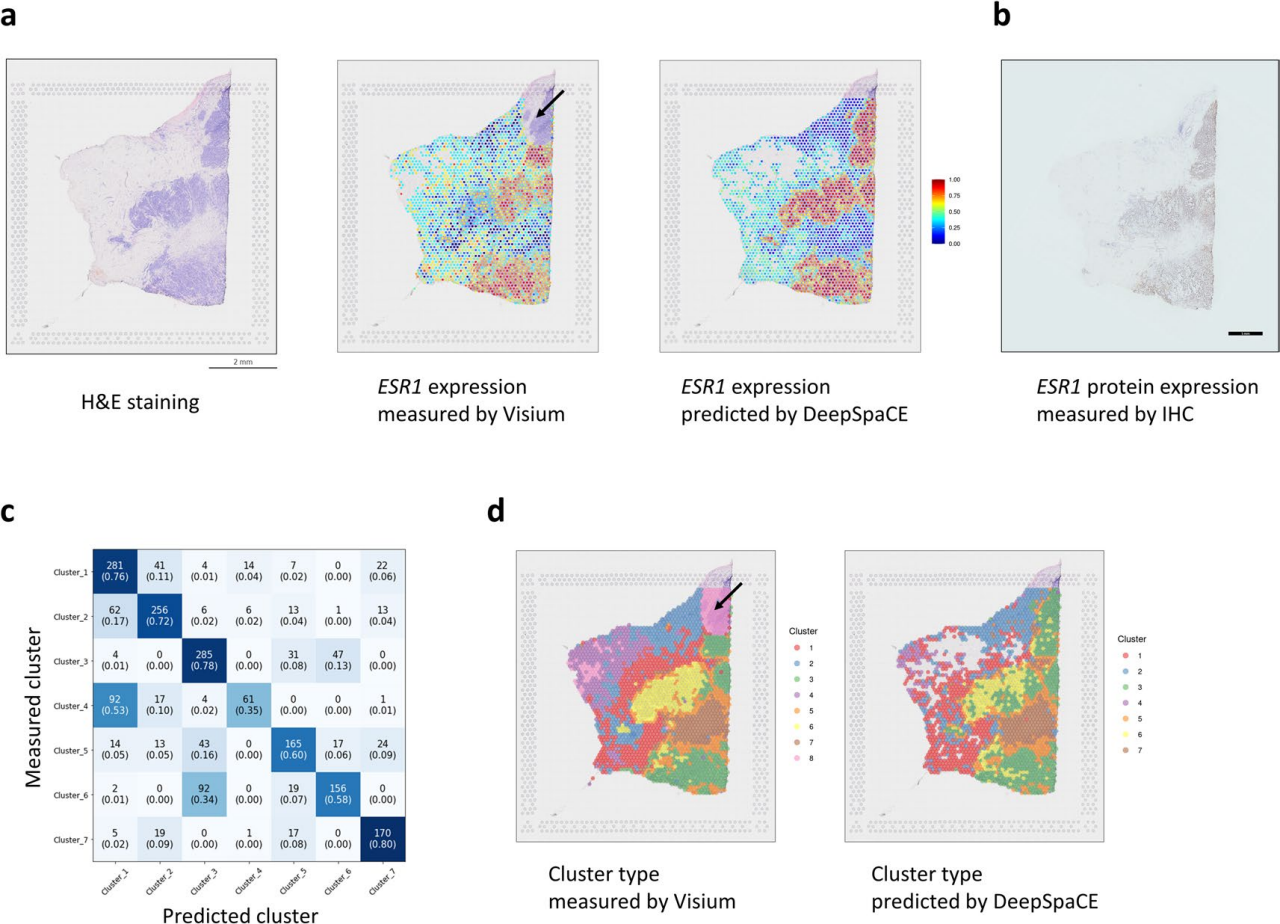
DeepSpaCE predicts spatial gene expression and cluster types. (**a**) Left image shows section D2 after hematoxylin and eosin (H&E) staining. Middle image shows a heatmap of normalized *ESR1* expression in section D2, measured using the 10x Genomics Visium platform. *ESR1* expression in the upper right region (black arrow) of section D2 could not be measured because of potential permeabilization errors. Right image shows the heatmap of *ESR1* expression in section D2, predicted by DeepSpaCE. The blank areas represent spots excluded because of a small amount of information. (**b**) Image showing immunohistochemical staining of ESR1 protein in the adjacent section of section D2. (**c**) Heatmap shows the confusion matrix of the measured and predicted cluster types. The number in each box is the number of labels, and the number inside each pair of parentheses is the recall value. (**d**) Left image shows a heatmap of transcriptomic cluster types in section D2, as measured using the Visium platform. The cluster types in the upper right region (black arrow) of section D2 were not determined because of potential permeabilization errors. Right image shows the heatmap of cluster types in section D2, predicted by DeepSpaCE. Blank areas represent spots that were excluded because of low information. For training and gene prediction parts, we excluded cluster 8 because most of the region belonging to the cluster showed potential permeabilization errors.

We trained the DeepSpaCE models of three breast cancer-marker genes, estrogen receptor 1 (*ESR1*), erb-b2 receptor tyrosine kinase 2 (*ERBB2*), and marker of proliferation Ki-67 (*MKI67*)^15,16^. We performed the fivefold cross-validation using randomly selected 80% spots of section D2 as training datasets and the remaining 20% spots of section D2 as testing datasets. The Pearson’s correlation coefficients between the measured and predicted values were 0.588 (standard deviation [SD] = 0.025; *ESR1*), 0.424 (SD = 0.050; *ERBB2*), and 0.219 (SD = 0.041; *MKI67*) (Supplementary Table S1, Supplementary Fig. S3). Notably, comparison of *ESR1* levels with H&E staining highlighted undetected highly expressed spots in the upper right region of section D2, possibly because of a potential permeabilization error in the Visium experiment (Fig. 2a). This observation was further confirmed by determining the protein-expression pattern observed by immunohistochemical staining of the adjacent section using an *ESR1* antibody (Fig. 2b). We quantified the immunohistochemistry image, and the predicted *ESR1* levels and estimated protein levels were well correlated (Pearson’s correlation coefficient: 0.600 (95% CI: 0.572–0.626)), as expected (Supplementary Fig. S4). Therefore, we concluded that the prediction of *ESR1* expression by DeepSpaCE was validated by immunohistochemistry, suggesting the applicability of our DeepSpaCE method for section imputation.

We performed multi-task learning of all genes (18,542 genes) in section C (Supplementary Fig. S5). The prediction accuracy of single-task and multi-task (21 genes/3 genes) were almost the same (Supplementary Table S2). The multi-task of all genes worked since the mean Pearson’s correlation coefficient was 0.129. However, the prediction accuracy of multi-task (all genes) was lower than that of multi-tasks (21 genes/3 genes). Multi-task learning effectively reduces training time, although the number of genes that we can include in multi-task learning of DeepSpaCE is limited.

We performed gene set enrichment analysis (GSEA)^17^ to characterize highly predictable genes. We used 18,451 genes expressed in section C, and tested the enrichment of hallmark gene sets in MSigDB^18^ (version 7.5) in the genes with higher Pearson's correlation coefficients between measured and predicted expression values. The most enriched hallmark gene set was “EPITHELIAL MESENCHYMAL TRANSITION” (EMT), whose description in MSigDB is “genes defining epithelial-mesenchymal transition, as in wound healing, fibrosis and metastasis” (Supplementary Table S3). *SPARC*, a gene well predicted by DeepSpaCE as shown below, was actually one of the leading-edge subsets (i.e., *SPARC* contributed the enrichment.) within the EMT gene set. Conversely, genes with low correlation coefficients did not show enrichment for any gene sets.

In addition, we applied DeepSpaCE for the other breast cancer tissues (Section E and F) obtained from a different patient from sections A–C and D1–D3 (Supplementary Fig. S6). Although the DeepSpaCE model was trained using section C, the model predicted the expression in sections E and F such as Pearson’s correlation coefficients of *ESR1* were 0.357 (SD = 0.014; section E) and 0.318 (SD = 0.009; section F) (Supplementary Table S4).

### Prediction and experimental validation of spatial transcriptomic cluster types

Based on the fivefold cross-validation in section D2, we assessed the prediction accuracy of transcriptomic cluster type derived from Space Ranger software. By comparing the clusters from Space Ranger with the predicted clusters, we first calculated the recall value (see “Methods”) of the clusters, which ranged from 35% (cluster 4) to 80% (cluster 7) (Fig. 2c). Although, there were mis-predictions when comparing non-cancerous regions (e.g., clusters 1 and 4), the cluster types between cancer sites and non-cancer sites were clearly distinguishable (e.g., clusters 1 and 3). We also calculated micro/macro-precision, recall, f1-score, and area under the curve (AUC) values (Supplementary Table S5). We concluded the prediction performance of DeepSpaCE was enough since macro-AUC was 0.937. Importantly, similar to the findings described in the previous section, the cluster type was predicted in the unmeasured upper right region of section D2, which showed a potential permeabilization error (Fig. 2d). The predicted clusters in this region were plausible based on the spatial transcriptome and DeepSpaCE analysis using the adjacent sections D1 and D3, which could measure the right upper region (Supplementary Fig. S7). We considered that each cluster of the right upper region were similar (Cluster 3 in section D1, Cluster 3 in section D2, and Cluster 6 in section D3) since the pathway analysis indicated that the Cluster 3 in section D1, Cluster 3 in section D2, and Cluster 6 in section D3 have the very similar components (e.g., estrogen-responsive cells) (Supplementary Table S6).

We performed pathway analysis of each cluster to clarify the relationship between cluster similarity and prediction error of DeepSpaCE in section D2. We showed the top 5 pathways of each cluster (Supplementary Table S7). Briefly, clusters 1, 2, 4, and 7 are well characterized as cells under epithelial-mesenchymal transition, clusters 3 and 6 are estrogen-responsive cells, and cluster 5 might be heterogeneous because the epithelial-mesenchymal transition is both upregulated and downregulated. These similarities based on pathways can explain the prediction errors. For example, the misprediction of Cluster 4 as Cluster 1 was relatively large (53%) (Fig. 2c). Cluster 4 and Cluster 1 were adjacent and similar tissue (microenvironment) in a section, and both clusters activated the same pathways (Epithelial-Mesenchymal Transition and UV Response Dn). The misprediction of Cluster 6 as Cluster 3 was also relatively large (34%). Cluster 6 and Cluster 3 were not adjacent, but similar tissue (tumor) activated the same pathways (Estrogen Response Early, Epithelial-Mesenchymal Transition, Allograft Rejection, Inflammatory Response, TNF-alpha Signaling via NF-kB, and Interferon Gamma Response). In summary, overall DeepSpaCE predicted the cluster types (max recall value: 80% (cluster 7)) although there is a limitation if the clusters are adjacent and/or biologically similar types of clusters (minimum recall value: 35% (cluster 4)).

### Super-resolution of spatial gene expression

We performed super-resolution for *ESR1* in the images of spots measured in section C (Supplementary Fig. S8a,b). Section C was used as both a training and test set to generate a super-resolved image as an example. The super-resolved image of *ESR1* expression in section C was consistent with the immunohistochemical staining results, supporting that DeepSpaCE enables accurate high-resolution observations of expression profiles (Supplementary Fig. S8c). Notably, we found a region with low *ESR1* expressions in the super-resolved image, which was not clearly observed in the original spatial transcriptome datasets (Supplementary Fig. S8b), confirming the importance of super-resolution.

**Figure 3:**
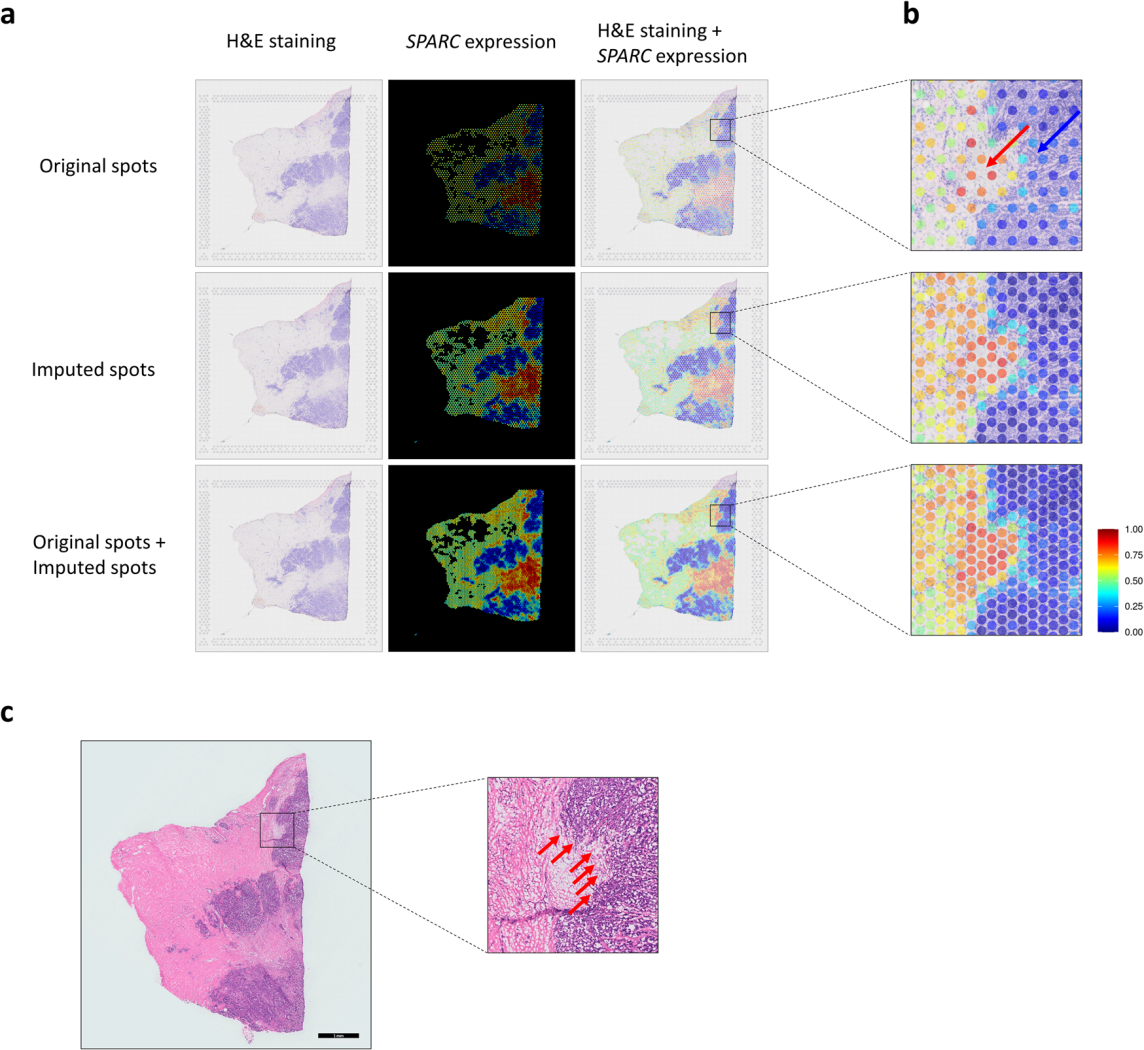
Super-resolution of *SPARC* expression as a practical application of DeepSpaCE. Super-resolution of *SPARC* expression using DeepSpaCE highlights tumor invasion more clearly and semi-supervised learning for tissue section imputation using DeepSpaCE improves prediction accuracy. (**a**) Nine images show the super-resolved results for *SPARC* expression. Three images in the left column show section D2 after H&E staining. Three images in the middle column show the heatmaps of predicted *SPARC* expression by DeepSpaCE for the original spots (top), imputed spots (middle), and both original and imputed spots (bottom). Three images in the right column show overlays of predicted *SPARC* expression by DeepSpaCE and H&E staining for section D2. (**b**) Three enlarged images on the right area show tumor cell invasion (blue arrow) and the microenvironment (red arrow). Spot size is adjusted to smaller than the exact spot size of the Visium platform to show the background image. (**c**) Left image shows the H&E-stained section adjacent to section D2. Right enlarged image is the same region as (**b**). Enlarged image shows the invasion of tumor cells. We drew annotated outlines of invasive regions in the H&E images. The red arrows show the invasive region.

We focused on secreted protein acidic and cysteine rich (*SPARC*), a potential cancer-invasion marker, and assessed whether the super-resolution method could facilitate biological interpretations provided by pathologists based on histology patterns in H&E-stained images. After training and validating the DeepSpaCE model, we predicted *SPARC*-expression levels among the original spots in section D2 (Fig. 3a,b). Although the color intensities themselves did not explain the *SPARC* expression patterns in the H&E-stained images, the patterns were successfully predicted by the DeepSpaCE model. For example, the *SPARC* expression levels in two circles on the bottom are quite different although it looks similar in color intensity (Supplementary Fig. S9). Comparison of the super-resolved image of *SPARC* with the H&E-stained image showed that the invasive tumor region overlapped substantially with the distribution of *SPARC* expression (Fig. 3c). In contrast, such patterns were not apparent from the color intensities in the original spatial-transcriptome data. Importantly, the spatial expression profiles of dense spots imputed by DeepSpaCE and their gene annotations enable delineating a functional boundary clearly. *SPARC* is secreted into the extracellular matrix from cancer and stromal cells, and high *SPARC*-mRNA expression is related to metastasis and poor prognosis in several types of cancers^19^. Thus, the super-resolved *SPARC*-expression image highlighted the potential tumor-invasion region and made it easier to identify by a non-pathologist in cases where a given transcript’s function is known. This is an intrinsic value of the super-resolution of DeepSpaCE.

### Imputation of a tissue section using semi-supervised learning

**Figure 4:**
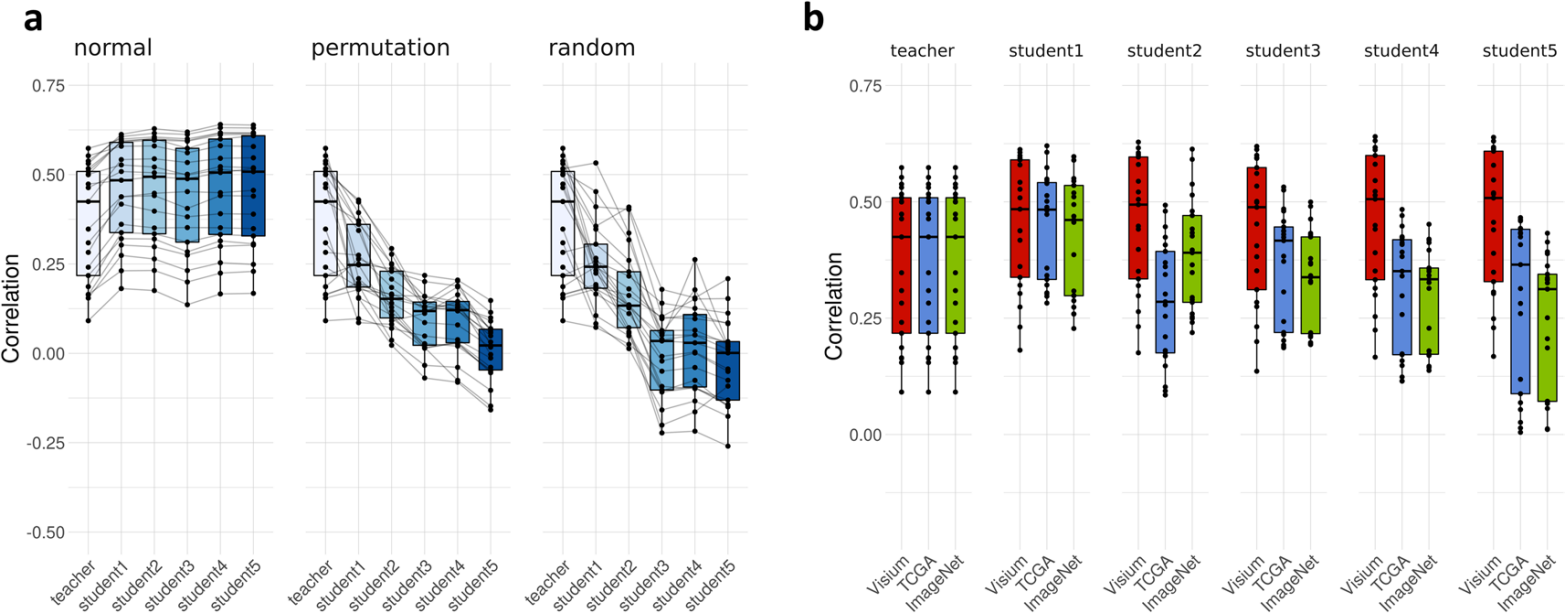
Semi-supervised learning for the tissue section imputation. (**a**) Box plots show Pearson’s correlation coefficients between the measured and predicted gene-expression levels of 21 breast cancer-related microenvironment markers. Left box plot displays the results of semi-supervised learning, which showed increasing Pearson’s correlation coefficients. Middle and right box plots show the semi-supervised learning results with permutated and randomized values. For the box plot, the box indicates the first and third quartiles; horizontal center line marks the medians; upper whisker extends from the hinge to the highest value that is within 1.5× interquartile range (IQR) of the hinge; lower whisker extends from the hinge to the lowest value within 1.5× IQR of the hinge; and data were plotted as points. Black lines between boxes connect the same gene. (**b**) Box plots show Pearson’s correlation coefficients between the measured and predicted gene-expression levels of 21 breast cancer-related microenvironment markers. Three types of image sets were compared for semi-supervised learning, namely sections A–C (red); data from The Cancer Genome Atlas (TCGA) (blue); and ImageNet data (green). For the box plot, the box indicates the first and third quartiles; horizontal center line marks the medians; upper whisker extends from the hinge to the highest value that is within 1.5× IQR of the hinge; lower whisker extends from the hinge to the lowest value within 1.5× IQR of the hinge; and data were plotted as points.

To assess whether semi-supervised learning can improve the prediction accuracy of DeepSpaCE, we performed tissue section imputation for section D2 using the model trained by sections D1 and D3 (randomly selected 80% spots were used as training and validating data and the others were used as test data; see “Methods”). We used the same test data for only evaluating both of the teacher model (first trained model) and student models (semi-supervised models). Thus, the test data set information was never used for the training and validation procedure. We performed semi-supervised learning as an option in DeepSpaCE. In using semi-supervised learning, unlabeled images were used to improve the prediction accuracy with predicted proxy labels using the noisy student method^20^. In section imputation, the teacher model was first trained with training data (sections D1 and D3). Next, the teacher model added the proxy labels to the unlabeled images (sections A–C). Then, the student 1 model was trained using both training data (sections D1 and D3) and unlabeled images (sections A–C) with proxy labels. The process is repeated until the student 5 model is trained. We selected the most predictive student model in the validation data as the best model. We found an increasing trend in the Pearson’s correlation coefficients between the measured and predicted expression levels (Supplementary Table S8). In the teacher model, the mean Pearson’s correlation coefficient for 21 genes was 0.369. The mean Pearson’s correlation coefficients progressively increased for student model 1 (0.414), student model 2 (0.455), student model 3 (0.438), student model 4 (0.457), and student model 5 (0.458) (Fig. 4a). For *SPARC*, Pearson’s correlation coefficient increased from 0.509 (SD = 0.069; teacher model) to 0.616 (SD = 0.067; student model 4, the best model for predicting *SPARC*). For *MXRA5*, the Pearson’s correlation coefficient was not increased after student model 1, although it was increased from 0.501 (SD = 0.083; teacher model) to 0.527 (SD = 0.093; student model 1). Therefore, the semi-supervised learning method may increase the accuracy of DeepSpaCE through additional computational costs.

To verify whether their related unlabeled images could improve the accuracy of the DeepSpaCE model, we performed semi-supervised learning with permutated gene-expression levels or randomized the values as negative controls. The Pearson’s correlation coefficients did not increase but decreased when permutated or randomized values were used. Moreover, Pearson’s correlation coefficients did not increase when irrelevant images of dogs or cats (obtained from ImageNet) were used for semi-supervised learning. Furthermore, Pearson’s correlation coefficients did not increase when breast cancer section images obtained from The Cancer Genome Atlas (TCGA) were used for semi-supervised learning (Fig. 4b).

We performed linear regression analyses to quantify how much DeepSpaCE explains the predicted expressions compared with the baseline model which is composed of simplified predictor such as average color channels (mean RGB values), brightness, and log(total UMIs) (see “Methods”). We show R-squared values of baseline and full model for predicting *SPARC* expression in testing data from the first iteration of the fivefold cross-validation, and the P-value of the coefficient for predicted expression level by DeepSpaCE in the full model (Table 1). In the teacher model, mean RGB values explained *SPARC* expression level better than DeepSpaCE, although DeepSpaCE explained *SPARC* expression level better than brightness and log(total UMIs). In the student 4 model that we proposed as the best model to predict, DeepSpaCE explained *SPARC* expression level better than all baseline models. In conclusion, our DeepSpaCE has a significantly higher prediction performance than classical simplified predictors such as average color channels of an image, especially using semi-supervised learning.

**Table 1:** Linear regression analysis.

Model	Predictor	Baseline model	Full model	Coefficient for DeepSpaCE		
		Adjusted R2	Adjusted R2	Beta	SE	P-value
Teacher model	Mean RGB	0.306	0.306	0.0736	0.328	0.822
	Brightness	0.220	0.245	2.45	0.295	1.98E−16
	log(totalUMI)	0.076	0.214	4.63	0.246	8.31E−73
Student model	Mean RGB	0.306	0.357	0.468	0.0371	4.55E−35
	Brightness	0.220	0.358	0.569	0.0274	8.04E−87
	log(totalUMI)	0.076	0.389	0.664	0.0207	6.86E−182

The table shows the adjusted R-squared value of the baseline model, adjusted R-squared value in the full model, and beta, SE, and P-value of coefficient for DeepSpaCE.

The values were calculated using mean RGB values, brightness, and log(total UMI) in the teacher model and student 4 model.

We also performed the cross-section validation of section imputation using sections D1–D3. The prediction accuracy of section D1 prediction (training with sections D2 and D3) (Supplementary Table S9) and section D3 prediction (training with sections D1 and D2) (Supplementary Table S10) were decreased prediction accuracy compared with section D2 prediction (training with sections D1 and D3). That is a reasonable result since section D2 was sandwiched with sections D1 and D3.

### Performance comparison with different methods

We compared VGG16 architecture with DenseNet121 used in ST-Net^21^ (Supplementary Table S11, Supplementary Fig. S10). In the case of the teacher model, the Pearson’s correlation coefficient of *SPARC* was higher in VGG16 (R = 0.450 (standard deviation [SD] = 0.172)) than DenseNet121 (R = 0.471 (SD = 0.066)). However, semi-supervised learning showed the higher correlation coefficients for *SPARC* (R = 0.613 (SD = 0.089), VGG16, student model 1). Thus, we kept VGG16-based results and set the default model for DeepSpaCE (DeepSpaCE users can change the model if necessary).

We performed XFuse^5^, which is the method to generate super-resolved transcriptomics images using generative models (Supplementary Fig. S11). We found that XFuse could not interpolate the potential permeabilization error region. In addition, we found that XFuse was not optimized for the Visium dataset because, in the training process, XFuse cannot consider the fiducial frame (region of four side’s dots) that Space Ranger needs to align tissue slide with barcoded spots. On the other hand, DeepSpaCE compensates for potential permeabilization error regions, and it was appropriately optimized for the Visium dataset. These are advantage points of DeepSpaCE over XFuse.

## Discussion

This study proposed performing super-resolution and section imputation with DeepSpaCE and validated the accuracy from cross-validation and immunohistostaining. These approaches made it possible to derive more knowledge from existing spatial transcriptome datasets. We performed GESA using Pearson's correlation coefficients between measured and predicted expression values to infer what types of genes were well predictable by DeepSpaCE. As a result, the EMT gene set, including *SPARC,* was most significant. Since EMT drastically changes the cell morphology of tumor cells and is a heterogeneous state^22,23^, the enrichment was considered quite reasonable because DeepSpaCE needs to be able to recognize morphological features to predict expression levels. Therefore, it suggested that DeepSpaCE can predict expression levels of heterogeneously expressed genes. The relationship between *SPARC* expression and cancer invasion was highlighted in super-resolution, whereas detecting the invasive region using original spatial transcriptome data was difficult because the measured spots were not dense (Fig. 3a,b). The *SPARC* glycoprotein has a high affinity for albumin, and macrophage-derived *SPARC* contributes to metastasis by acting at the step of integrin-mediated migration of invasive cells^19^. Previously, *SPARC* mRNA expression was reported as a predictor of a pathological complete response after neoadjuvant nab-paclitaxel therapy^24^. Our study underscored the relationship between *SPARC* expression and invasive regions, which may be clinically important for treating breast cancer. This interpretation does not require expertise in histology or pathology but gene annotations, which spatial transcriptome researchers should be familiar. In addition, super-resolution in section C identified the region with low expressed *ESR1*, the amplification of which is frequently observed in proliferative breast cancers^15^. Although it is unclear whether the region indicates the heterogeneity of breast cancer tissues or the existence of normal tissues, this region was unclear in the original spatial transcriptome data and experimentally validated expression in adjacent sections.

For super-resolution and section imputation, we developed DeepSpaCE to predict expression levels from spot images from Visium. This spot-level analysis should reveal more detailed patterns than those obtained by CNN using pairs of images of the bulk transcriptome^25^ by resolving spatial expression patterns. DeepSpaCE requires a minimum of a single experiment to analyze the spatial transcriptome; nevertheless, the predictions were well-validated by cross-validation and experimental analysis. DeepSpaCE as well as super-resolution and section imputation methods aim to maximize the value of existing datasets and provide foundations for subsequent experiments from at least a single dataset without additional experimental costs. This attribute is an important difference from the recently proposed ST-Net study where trained spatial transcriptome data (not from the Visium platform) was obtained from as many as 23 individuals^21^.

The number of training datasets used for single spatial transcriptome analysis (maximum 4992 spots/slide with the Visium platform) was insufficient for training the CNN in general, as a previous study used ~ 557,000 images from 830 slides to predict lung cancer subtypes and ~ 212,000 images from ~ 320 slides to predict lung cancer gene mutations^11^. To increase the ability to apply DeepSpaCE to many datasets for which it is challenging to train the connections between H&E-stained images and expression levels, we implemented a semi-supervised learning method^26^ in DeepSpaCE. The DeepSpaCE model with semi-supervised learning using sections A–C as unlabeled images showed better performance than a simple prediction model using only experimentally obtained spatial transcriptome data. Although we increased the predictive accuracy of tissue section imputation in this case, the Pearson’s correlation coefficients were not improved when using breast cancer H&E-stained images obtained from TCGA as unlabeled images. This result may be because the DeepSpaCE model is sensitive to the protocol for obtaining the H&E images (i.e., batch effects disturb the training steps). We performed semi-supervised learning for cluster type prediction (Supplementary Table S12). Although prediction accuracy was not improved by semi-supervised learning, the AUC was sufficiently high in the teacher model as we described (Supplementary Table S5), suggesting that there is not enough room to improve the accuracy in this task. Therefore, the model that gives the best prediction accuracy should be determined when using semi-supervised learning as an option.

As an experimental validation, we confirmed that predicted *ESR1* expression levels were correlated with protein levels quantified from the immunohistochemistry image (Supplementary Fig. S4). However, RNA levels generally do not entirely correlate with protein levels due to post-transcription events^27^. This is a limitation of our additional experimental validation for DeepSpaCE.

Although overcoming the domain shift problem is important but challenging, DeepSpaCE aims to obtain super-resolved spatial transcriptomic profiles within a single slide and imputing the intersection of consecutive sections (Fig. 1). Therefore, DeepSpaCE expects the data is derived from a single domain, with images from the same scanner and spatial transcriptomic data measured by the same protocol. Nevertheless, we increased the variability of images by using image augmentation to avoid potential domain shift, and they improved the Pearson’s correlation coefficients (Supplementary Fig. S14). Furthermore, we confirmed that the model trained with one patient worked well to a section image derived from a different patient (Supplementary Table S4).

Even if a high-resolved spatial transcriptome technology will be released in the future, the value of DeepSpaCE will not be diminished because DeepSpaCE can impute the section in consecutive sections (Fig. 4). It reduces the experimental costs and clarifies biological functions at higher resolution and in three dimensions. Also, the higher resolution spots in a slide might need more sequencing costs for getting the same number of detected genes. We also consider that DeepSpaCE can apply to other spatial transcriptomic platforms such as GeoMX^28^ because our DeepSpaCE method just needs H&E image(s) and the corresponding spatial transcriptome datasets. Although slight code modification would be required for matching formats of GeoMX’s input datasets, users can use our core python scripts for running DeepSpaCE under GNU General Public License v3.0. In conclusion, DeepSpaCE is an all-in-one package that augments spatial transcriptome data obtained from the in situ capturing platform; its applications can improve the understanding of histological expression profiles.

## Methods

### Ethical approval

Breast tissue samples and relevant clinical data were obtained from patients undergoing surgery at St. Marianna University School of Medicine Hospital after obtaining approval from the Clinical Ethics Committee of St. Marianna University (approval number: 2297-i103). The approval allowed the retrieval of surgical pathology tissues that were obtained with informed consented or that were approved for use with a waiver of consent. All methods were performed in accordance with the relevant guidelines and regulations.

### Spatial-transcriptomics datasets

We used six human breast cancer tissue sections, including sections A–C and consecutive sections D1–D3, which were derived from one patient. We also used two breast cancer tissues (sections E and F) obtained from a different patient. The spatial transcriptomics experiments were conducted with the same protocol reported in Nagasawa et al.^8^. Briefly, the tissue sections were stained with H&E, and TIFF images were obtained using a microscope at 10× magnification. Spatial-transcriptome profiling was performed using the Visium platform with the standard protocol provided by 10× Genomics (Pleasanton, CA, USA). UMI counts were calculated using 10x Genomics Space Ranger software (version 1.0.0). Visium demo data (version 1.0.0) for the human heart tissue was obtained from the 10x Genomics website (https://www.10xgenomics.com/resources/datasets/).

### Preprocessing of spatial gene-expression data

Regarding the spatial-transcriptome profiles obtained from the Space Ranger pipeline (10x Genomics), we removed spots with low total UMI counts (< 1000) or a low number of measured genes (< 1000). The SCTransform function of Seurat package (version 3.1.4)^29^ was applied to normalize the UMI counts, based on regularized negative binomial regression^30^. Min–max scaling was performed to adjust the expression values between zero and one. We trained 24 genes including three breast cancer-marker genes (*MKI67*, *ESR1*, *ERBB2*) and 21 breast cancer-related microenvironment marker genes (*SPARC*, *IFI27*, *COL10A1*, *COL1A2*, *COL3A1*, *COL5A2*, *FN1*, *POSTN*, *CTHRC1*, *COL1A1*, *THBS2*, *PDGFRL*, *COL8A1*, *SULF1*, *MMP14*, *ISG15*, *IL32*, *MXRA5*, *LUM*, *DPYSL3*, and *CTSK*). These 21 genes were manually selected from the cluster of genes overexpressed in the breast cancer-related microenvironment region. These two gene sets of three genes and 21 genes were respectively used in the training part of DeepSpaCE. We removed low expressed genes (< 10%) for multi-task learning of all genes and used 18,542 genes in section C. The graph-based clustering algorithm^31^ implemented in Space Ranger was used for transcriptomic cluster type prediction. We separately performed clustering of sections D1–D3 since we used the output of Space Ranger in the default settings, considering users who are not bioinformaticians.

### Preprocessing of tissue section images

Each spot image was cropped from a tissue slide image, based on the position table in the Space Ranger outputs (Supplementary Table S13). As shown below, we filtered out whitish images in which more than half of the pixels were the > 80% percentiles of mean RGB values. For image augmentation, we randomly applied image-transform functions of flipping (RandomRotate90, Flip, and Transpose), cropping (RandomResizedCrop), noise (IAAAdditiveGaussianNoise and GaussNoise), blurring (MotionBlur, MedianBlur, and Blur), distortion (OpticalDistortion, GridDistortion, IAAPiecewiseAffine, and ShiftScaleRotate), contrast (RandomContrast, RandomGamma, and RandomBrightness), and color-shifting (HueSaturationValue, ChannelShuffle, and RGBShift) in Albumentations library (version 0.4.5)^32^.

### Preprocessing of images obtained from TCGA and ImageNet

We obtained 1978 images of H&E-stained TCGA breast cancer sections from the GDC Data Portal (https://portal.gdc.cancer.gov) on August 05, 2020. As negative controls, we obtained 14,500 irrelevant images such as dogs and cats (n02106662, n02110341, n02116738, n02123045, n02123159, n02123394, n02123597, n02124075, n02497673, and n03218198) from ImageNet (http://www.image-net.org) on October 09, 2020. All images obtained from TCGA and ImageNet were cropped to 224 × 224 pixels (Supplementary Fig. S12). Four thousand cropped images were randomly selected as unlabeled images for each semi-supervised learning model.

### Training and prediction of spatial gene-expression profiles and transcriptomic cluster types

All deep-learning models were implemented using the deep-learning framework PyTorch (version 1.5.1)^33^. We adapted the VGG16 architecture for the deep CNN model with 16 weight layers^13^. We modified the number of output features in VGG16 from 1000 to the number of genes or cluster types. We simultaneously trained multi genes such as three breast cancer markers or 21 breast cancer-related microenvironment markers. We also performed multi-task learning of all genes (18,542 genes) in section C (training and test sets). For transcriptomic cluster type predictions, the loss value of the training DeepSpaCE dataset was calculated using the CrossEntropyLoss function. For gene-expression predictions, the loss value was determined as the sum of loss calculated with the SmoothL1Loss function for each gene. As an optimizer, we used Adam^34^ with the hyperparameters of learning rate: 1e−4 and weight decay: 1e−4. Each training was repeated for 50 epochs to stabilize the loss curves (Supplementary Fig. S13). Early stopping was applied if the loss value for the validation data did not decrease over five continuous epochs. To evaluate the accuracy of cluster type prediction, we used the recall value which reflects the proportion of positives identified correctly among the actual number of positives (recall = true positive/(true positive + false negative)). We also calculated micro/macro-precision, recall, f1-score, and AUC values. The micro values were calculated for each cluster (i.e., the classification accuracy of a cluster vs. other clusters). The macro values are the mean values of the micro values. For section D2, cluster 8 was excluded from the training set because it consists of a region of potential permeabilization errors. We performed fivefold cross-validation to evaluate DeepSpaCE. In the fivefold cross-validation, we first separated the 20% of the dataset as a test set (20%) and used 20% of the remaining set as a validation set and others as a training set. The validation set was used for monitoring the progress of training and the decision to apply early stopping. The test set was only used for the final quantification of performance. In summary, we used completely separated three data sets, training set (64%), validation set (16%), and test set (20%) for validating DeepSpaCE. We showed the details of the training set, validation set, and test set which used for the super resolution and the section-imputation (Supplementary Table S14).

### Parameter optimization of DeepSpaCE

We optimized the parameters of DeepSpaCE, such as the image size, image-filtering threshold, and image-augmentation methods. We performed fivefold cross-validation using six sections (A–C, and D1–D3) to evaluate the prediction accuracy. We developed prediction models for the expression levels of the 24 genes including three breast cancer-marker genes (*MKI67*, *ESR1*, *ERBB2*) and 21 breast cancer-related microenvironment marker genes (described above) because these genes are representative markers of heterogeneous ductal carcinoma tissues. First, we assessed the impact of the size of the input images (0%, 50%, 100%, 150%, and 200%; relative to the original spot image size) on the prediction accuracies; the results showed that an image size of 150% gave better outcomes than the original and smaller image sizes (Supplementary Fig. S14a). This result is biologically plausible because the surrounding cells can communicate with cells in the spot and affect their gene-expression levels. Second, we assessed the different image-filtering thresholds to exclude uninformative images (i.e., excluding almost white images). We calculated whiteness for each spot by calculating the mean RGB values and obtained the percentiles (50%, 60%, 70%, 80%, 90%, and 100%) over spots in a slide. We filtered out images where more than half of the pixels were the > 80% percentiles of mean RGB values as judged from the histogram (Supplementary Fig. S14b). This strategy maximized the prediction accuracy (Supplementary Fig. S14c). Third, to further improve accuracy, we augmented images with various image transformations such as flipping, cropping, blurring, distortion, noise, contrast, and color-shifting (Supplementary Fig. S15). All image augmentation (except for color-shifting) improved the Pearson’s correlation coefficients compared with non-augmented images (Supplementary Fig. S14d); however, we also used the color-shifting method because H&E-staining on different slides may change the color intensities.

### Pathway analysis of transcriptomic clusters

To annotate each transcriptomic cluster in a section, we obtained differentially expressed genes between the cluster and others (positive (Fold-change > 0) or negative (Fold-change < 0); adjusted P-value < 0.05 (Benjamini–Hochberg method)) from Space Ranger in the default settings, and performed pathway analysis for them using Enrichr (Gene set: MSigDB Hallmark 2020)^35,36^. We considered pathways with adjusted P-value < 0.05 (Benjamini–Hochberg method) for each analysis as significant and summarized them (Supplementary Table S15).

### Gene set enrichment analysis of Pearson's correlation coefficients

We performed gene set enrichment analysis (GSEA)^17^ to interpret what types of genes were well predictable by DeepSpaCE using GSEA software (v4.1.0, Broad Institute). We used 18,451 genes expressed in section C which contains both tumor and microenvironmental regions and tested the enrichment of hallmark gene sets in MSigDB^18^ (Hallmark.all.v7.5) in the genes with higher Pearson's correlation coefficients between measured and predicted expression values.

### Super-resolution of spatial gene expression

New spot image files were created by cropping around three adjacent spots to impute the expression levels among spots on a slide image (Supplementary Fig. S16). We used sections C and D2 as both the training and test sets (randomly selected 80% spots were used as training data and others were used as test data). By performing super-resolution, the numbers of spots increased from 2238 to 6733 and from 2168 to 6623 in sections C and D2, respectively. We trained both of three breast cancer-marker genes and 21 breast cancer-related microenvironment marker genes, respectively. Semi-supervised learning was not used for super-resolution.

### Imputation of a tissue section using semi-supervised learning

Sections D1–D3 were obtained as consecutive sections. Thus, sections D1 and D3 were used as the training set. Section D2 was used as the test set to impute gene-expression levels because it was located between sections D1 and D3. Sections A–C were used for semi-supervised training as unlabeled images. In the noisy student model^20^, gene-expression levels in unlabeled images were predicted using the first trained model (teacher model). Four thousand predicted proxy labels and the associated images were added to the original dataset and used to train the next model, designated as a student model. The training student models were run five times (Supplementary Fig. S17a). In addition to the spot images of sections A–C, we used images of breast cancer sections obtained from TCGA as unlabeled images. Irrelevant images obtained from ImageNet were used as negative controls during semi-supervised learning. In addition, we also performed semi-supervised learning with permuted gene expression and random values as negative controls (Supplementary Fig. S17b).

### Linear regression analysis comparing with the baseline model

We performed linear regression analyses of average color channels (mean RGB values), the brightness of the image, and log(total UMIs) as the baseline model. We also performed linear regression analyses of the full model, which incorporated predicted expression level by DeepSpaCE to the baseline model. The formulas for the baseline and full model are shown below. We calculated R-squared values of baseline and full model for predicting *SPARC* expression in testing data from the first iteration of the fivefold cross-validation. We also calculated the P-value for the coefficient of predicted expression by DeepSpaCE in the full model. All analyses were performed by R (version 3.6.0).

Baseline model:

$$\left( {\text{Actual expression}} \right) \, \sim \, \left( {{\text{mean RGB}},{\text{ brightness}},{\text{ or log}}\left( {\text{total UMIs}} \right)} \right)$$

Full model:

$$\left( {\text{Actual expression}} \right) \, \sim \, \left( {{\text{mean RGB}},{\text{ brightness}},{\text{ or log}}\left( {\text{total UMIs}} \right)} \right) \, + \, \left( {\text{predicted expression level by DeepSpaCE}} \right)$$

### Performance comparison with different methods

In DeepSpaCE, we used VGG16^13^ as a deep learning model because the model is widely used as a gold standard (number of citations: 65,552, Google Scholar, October 24, 2021). We compared VGG16 with DenseNet121^37^ which was used in ST-Net. We performed XFuse with default parameters (below) in sections D1–D3. The parameters were network_depth: 6, network_width: 16, min_counts: 50, batch_size: 3, epochs: 100,000, learning_rate: 0.0003, and patch_size: 768.

### Immunohistochemistry and measurement of protein expression

Breast cancer tissues were frozen and embedded in an optimal cutting temperature compound (Sakura Finetek, Tokyo, Japan). Ten-micrometer-thick sections were cut onto slides using a Leica CM3050 S cryostat (Wetzlar, Germany), fixed in methanol at − 20 °C for 20 min, and air-dried for 60 min. Endogenous peroxidase activity was blocked in phosphate-buffered saline containing 3% H_2_O_2_ for 5 min. For *ESR1* staining, the sections were incubated with an anti-ESR1 antibody (FLEX Monoclonal Rabbit Anti-Human Estrogen Receptor α, Clone EP1, Catalog No. IS084, Agilent technologies, Dako, Glostrup, Denmark) at a 1:2 dilution for 60 min at room temperature. Antibody labeling was detected with the Histofine Simple Stain, MULTI (Nichirei Bioscience, Tokyo, Japan) following the manufacturer’s protocol, and all sections were counterstained with H&E. To quantify the immunohistochemistry image, we calculated the mean RGB values of each spot (14 × 14 pixels) on the IHC image, which implies the estimated protein levels (high mean RGB values indicate low protein levels, and vice versa).

### Data processing and analysis

Python (version 3.6.5) was used for preprocessing and implementation of DeepSpaCE with the libraries, torch (version 1.5.1), torchvision (version 0.6.1), numpy (version 1.19.0), pandas (version 1.0.5), scikit-learn (version 0.23.1), mlxtend (version 0.17.2), albumentations (version 0.4.5), opencv-python (version 4.2.0.34), and matplotlib (version 3.2.2). R (version 3.6.0) was used for statistical analysis and visualization with the packages, dplyr (version 1.0.2), data.table (version 1.12.8), Matrix (version 1.2.17), grid (version 3.6.0), rjson (version 0.2.20), hdf5r (version 0.9.7), readbitmap (version 0.1.5), ggplot2 (version 3.3.0), hrbrthemes (version 0.8.0), ggsci (version 2.9), ggpubr (version 0.4.0), cowplot (version 1.0.0), and Seurat (version 3.1.4.9904).

## Supplementary Information

Supplementary Information 1.

Supplementary Information 2.

Supplementary Information 3.

## Data Availability

All sequencing data and pathological images for Visium have been deposited in the DNA Data Bank of Japan under the accession number JGAS000202 and JGAS000290.

## Code availability

All codes for DeepSpaCE are available on GitHub (https://github.com/tmonjo/DeepSpaCE). These codes include image-preprocessing procedures and expression data produced from Space Ranger.

## References

[1] Ståhl PL (2016). Visualization and analysis of gene expression in tissue sections by spatial transcriptomics. Science.

[2] Rodriques SG (2019). Slide-seq: A scalable technology for measuring genome-wide expression at high spatial resolution. Science.

[3] Maniatis S (2019). Spatiotemporal dynamics of molecular pathology in amyotrophic lateral sclerosis. Science.

[4] Marx V (2021). Method of the year: Spatially resolved transcriptomics. Nat. Methods.

[5] Bergenstråhle L (2021). Super-resolved spatial transcriptomics by deep data fusion. Nat. Biotechnol..

[6] Thrane K, Eriksson H, Maaskola J, Hansson J, Lundeberg J (2018). Spatially resolved transcriptomics enables dissection of genetic heterogeneity in stage III cutaneous malignant melanoma. Cancer Res..

[7] Berglund E (2018). Spatial maps of prostate cancer transcriptomes reveal an unexplored landscape of heterogeneity. Nat. Commun..

[8] Nagasawa S (2021). Genomic profiling reveals heterogeneous populations of ductal carcinoma in situ of the breast. Commun. Biol..

[9] Yoosuf N, Navarro JF, Salmén F, Ståhl PL, Daub CO (2020). Identification and transfer of spatial transcriptomics signatures for cancer diagnosis. Breast Cancer Res..

[10] Asp M, Bergenstråhle J, Lundeberg J (2020). Spatially resolved transcriptomes—Next generation tools for tissue exploration. BioEssays.

[11] Coudray N (2018). Classification and mutation prediction from non–small cell lung cancer histopathology images using deep learning. Nat. Med..

[12] Nasrollahi K, Moeslund TB (2014). Super-resolution: A comprehensive survey. Mach. Vis. Appl..

[13] Simonyan, K. & Zisserman, A. *Very Deep Convolutional Networks for Large-scale Image Recognition**arXiv* http://arxiv.org/abs/1409.1556 (2015).

[14] 10x Genomics. *Visium Spatial Gene Expression Reagent Kits—Tissue Optimization*. 1–69 (2020).

[15] Holst F (2007). Estrogen receptor alpha (ESR1) gene amplification is frequent in breast cancer. Nat. Genet..

[16] Goldhirsch A (2013). Personalizing the treatment of women with early breast cancer: Highlights of the st gallen international expert consensus on the primary therapy of early breast Cancer 2013. Ann. Oncol..

[17] Subramanian A (2005). Gene set enrichment analysis: A knowledge-based approach for interpreting genome-wide expression profiles. Proc. Natl. Acad. Sci. USA..

[18] Liberzon A (2015). The molecular signatures database hallmark gene set collection. Cell Syst..

[19] Sangaletti S (2008). Macrophage-derived SPARC bridges tumor cell-extracellular matrix interactions toward metastasis. Cancer Res..

[20] Xie, Q., Luong, M. T., Hovy, E. & Le, Q. V. *Self-training with Noisy Student Improves Imagenet Classification* 10684–10695 (2020).

[21] He B (2020). Integrating spatial gene expression and breast tumour morphology via deep learning. Nat. Biomed. Eng..

[22] Sangaletti S (2016). Mesenchymal transition of high-grade breast carcinomas depends on extracellular matrix control of myeloid suppressor cell activity. Cell Rep..

[23] Ribatti D, Tamma R, Annese T (2020). Epithelial-mesenchymal transition in cancer: A historical overview. Transl. Oncol..

[24] Nakazawa Y (2020). The pathological complete response and secreted protein acidic and rich in cysteine expression in patients with breast cancer receiving neoadjuvant nab-paclitaxel chemotherapy. Oncol. Lett..

[25] Schmauch B (2020). A deep learning model to predict RNA-Seq expression of tumours from whole slide images. Nat. Commun..

[26] Ouali, Y., Hudelot, C. & Tami, M. *An Overview of Deep Semi-supervised Learning* arXiv http://arxiv.org/abs/2006.05278 (2020).

[27] Vogel C, Marcotte EM (2012). Insights into the regulation of protein abundance from proteomic and transcriptomic analyses. Nat. Rev. Genet..

[28] Merritt CR (2020). Multiplex digital spatial profiling of proteins and RNA in fixed tissue. Nat. Biotechnol..

[29] Stuart T (2019). Comprehensive integration of single-cell data. Cell.

[30] Hafemeister C, Satija R (2019). Normalization and variance stabilization of single-cell RNA-seq data using regularized negative binomial regression. Genome Biol..

[31] Blondel VD, Guillaume JL, Lambiotte R, Lefebvre E (2008). Fast unfolding of communities in large networks. J. Stat. Mech. Theory Exp..

[32] Buslaev A (2020). Albumentations: Fast and flexible image augmentations. Information.

[33] Stevens E, Antiga L, Viehmann T (2020). Deep learning with PyTorch.

[34] Kingma, D. P. & Ba, J. L. *Adam: A Method for Stochastic Optimization* http://arxiv.org/abs/1412.6980 (2015).

[35] Chen EY (2013). Enrichr: Interactive and collaborative HTML5 gene list enrichment analysis tool. BMC Bioinform..

[36] Kuleshov MV (2016). Enrichr: A comprehensive gene set enrichment analysis web server 2016 update. Nucleic Acids Res..

[37] Huang, G., Liu, Z., Van Der Maaten, L. & Weinberger, K. Q. Densely connected convolutional networks. In *Proc.—30th IEEE Conf. Comput. Vis. Pattern Recognition, CVPR 2017* 2017-Janua, 2261–2269 (2017).

