# Supplementary material for: Efficient prediction of a spatial transcriptomics profile better characterizes breast cancer tissue sections without costly experimentation
Source: Sci Rep. 2022 Mar 8;12:4133. doi: 10.1038/s41598-022-07685-4 (PMC8904587; doi:10.1038/s41598-022-07685-4)
Supplement: Supplementary file 1 — Supplementary Information 1. [file 41598_2022_7685_MOESM1_ESM.docx]

**Supplementary Information**

**
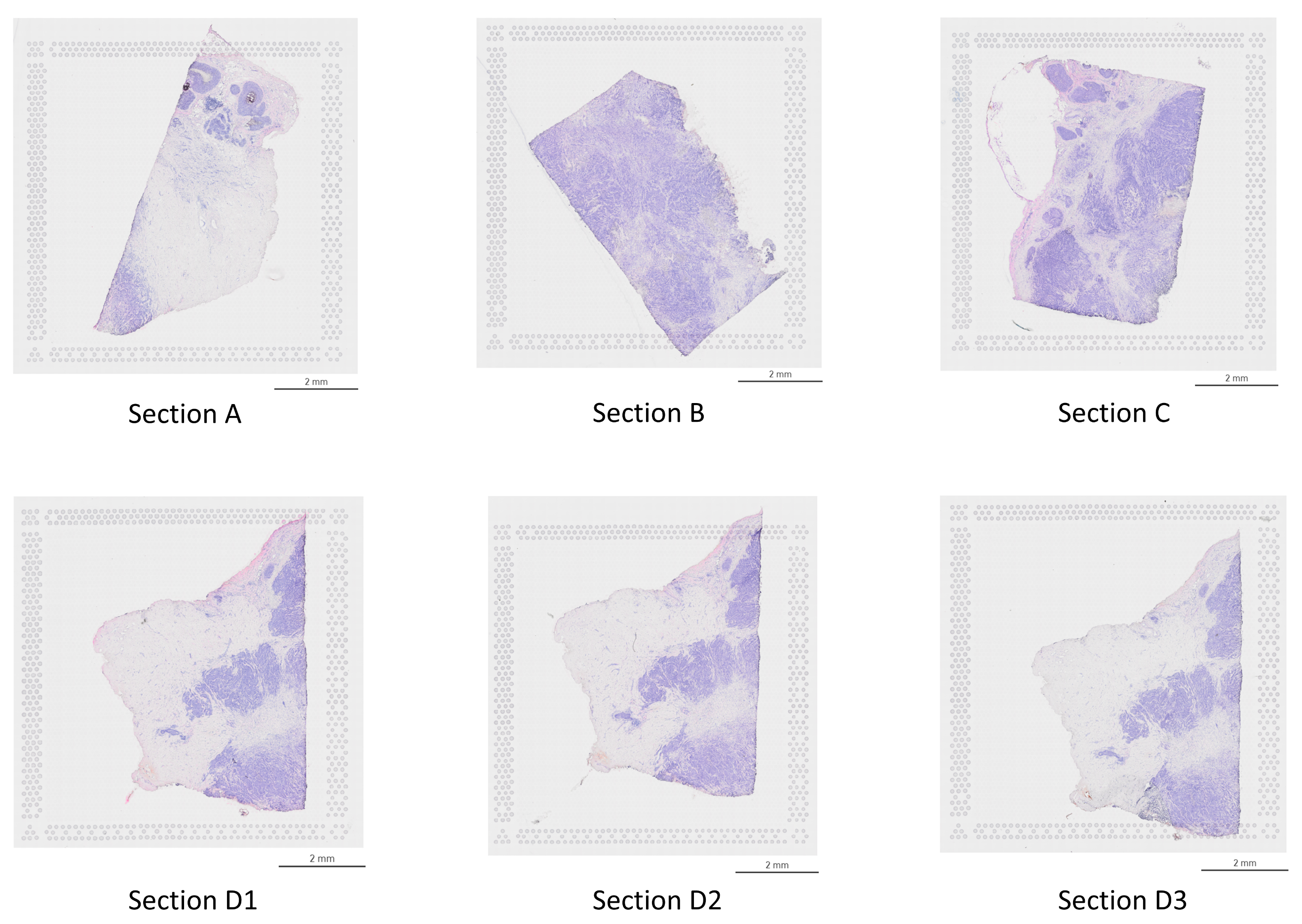
**

# **Supplementary Figure S1** Human breast cancer tissue sections (Sections A–C and D1–D3). Six slide images show H&E-stained human breast cancer tissues from sections A–C and consecutive sections D1–D3. Sections A–C and D1–D3 were derived from one patient.


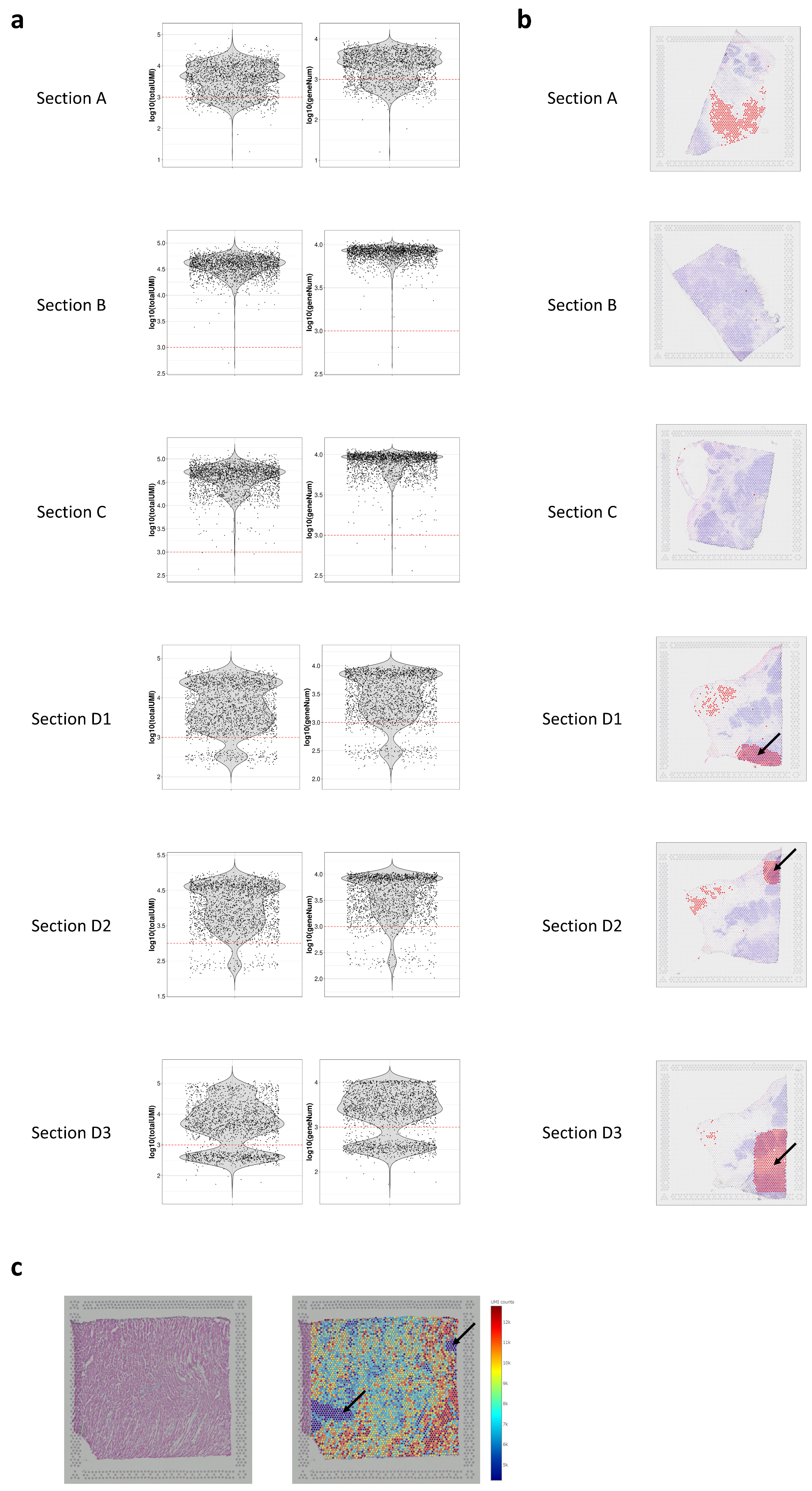


# **Supplementary Figure S2** Gene-expression normalization and filtering. (**a**) Violin plot shows the log10 transformed total unique molecular identifier (UMI) counts (left) and number of measured genes (right) in sections A–C and D1–D3. The spots under the red line were filtered out. For the violin plot, the red horizontal line is the threshold of spot filtering, and the data were plotted as points. (**b**) Heatmaps overlaid on H&E-stained sections showed that the red spots were removed because of low UMI counts (or the number of measured genes) in sections A–C and D1–D3. The right bottom regions in sections D1 and D3, as well as the right upper region in section D2, showed undetected regions because of potential permeabilization error (black arrows) (**c**) Left image shows the H&E-stained section of human heart tissue obtained from 10x Genomics website. The right image shows the heatmap of total UMI counts measured by Visium in the human heart tissue section. The heatmap image was obtained from Space Ranger. Total UMI counts were extremely low in the upper right and the lower left region of the section (black arrows), likely because of a technical problem such as permeabilization error.


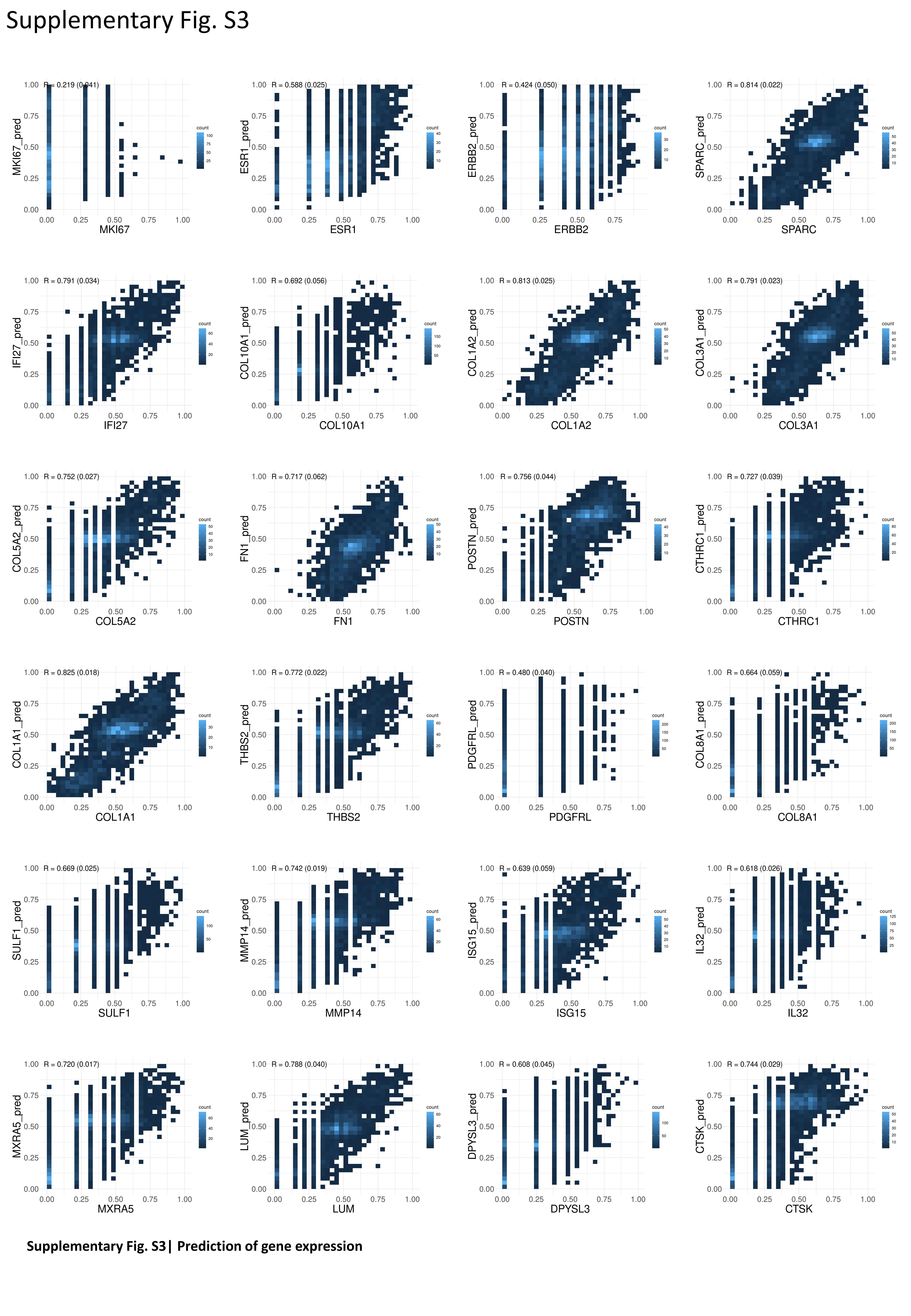


# **Supplementary Figure S3** Prediction of gene expression. The plots show the expression values obtained from the 5-fold cross-validation in section D2. 24 plots are including three breast cancer-marker genes (*MKI67*, *ESR1*, and *ERBB2*) and 21 breast cancer-related microenvironment marker genes. The x-axis is the measured expression value, and the y-axis is the predicted value. The intensity of the color indicates the number of dots. The upper left value is the mean Pearson’s correlation coefficients of 5-fold cross-validation, and the number inside each pair of parentheses is the standard deviation.

**
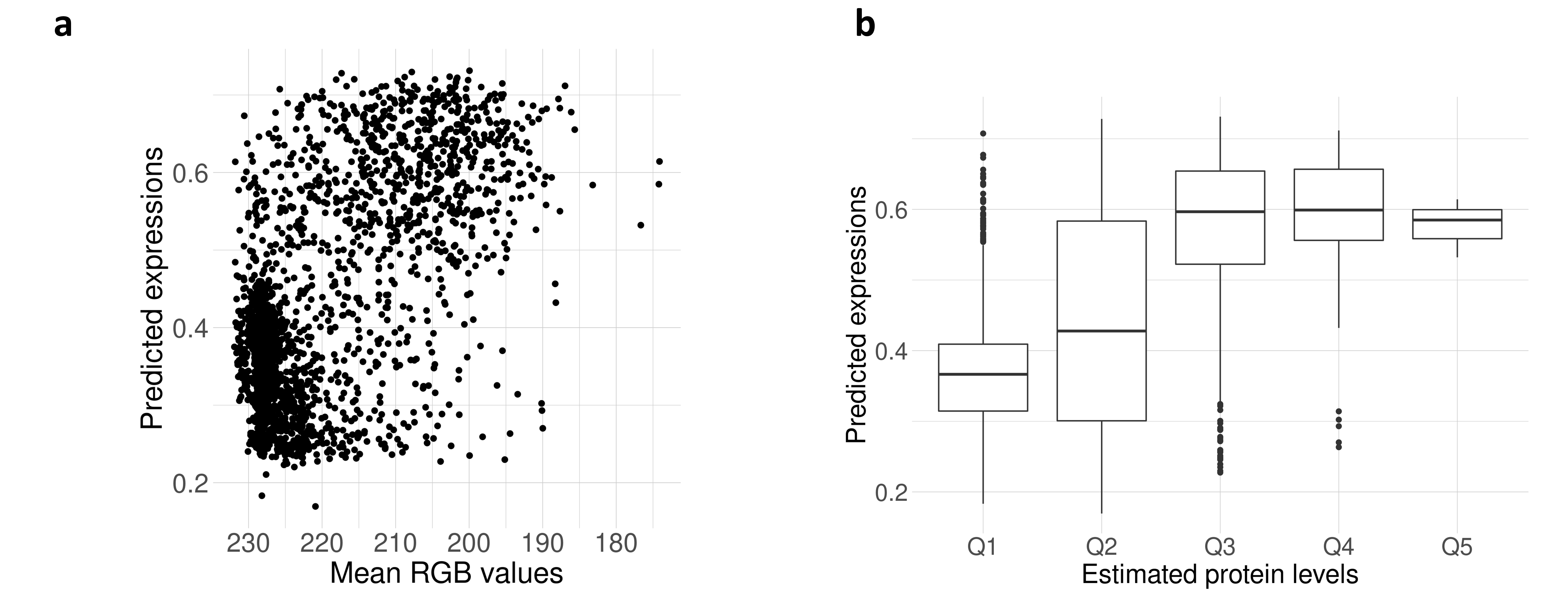
**

# **Supplementary Figure S4** Quantitative analysis of the immunohistochemistry image. (**a**) The scatter plot shows the result of quantitative analysis of the immunohistochemistry image of **Fig. 2b**. The x-axis is the mean RGB values, and the y-axis is the *ESR1* expression levels predicted by DeepSpaCE. (**b**) The boxplot shows the predicted *ESR1* expression on the y-axis and inferred protein level (reverse mean RGB values) on the x-axis. The estimated protein levels mean the mean RGB values which were split into five bins; Q1: (225,240], Q2: (210,225], Q3: (195,210], Q4: (180,195], and Q5: [165,180].


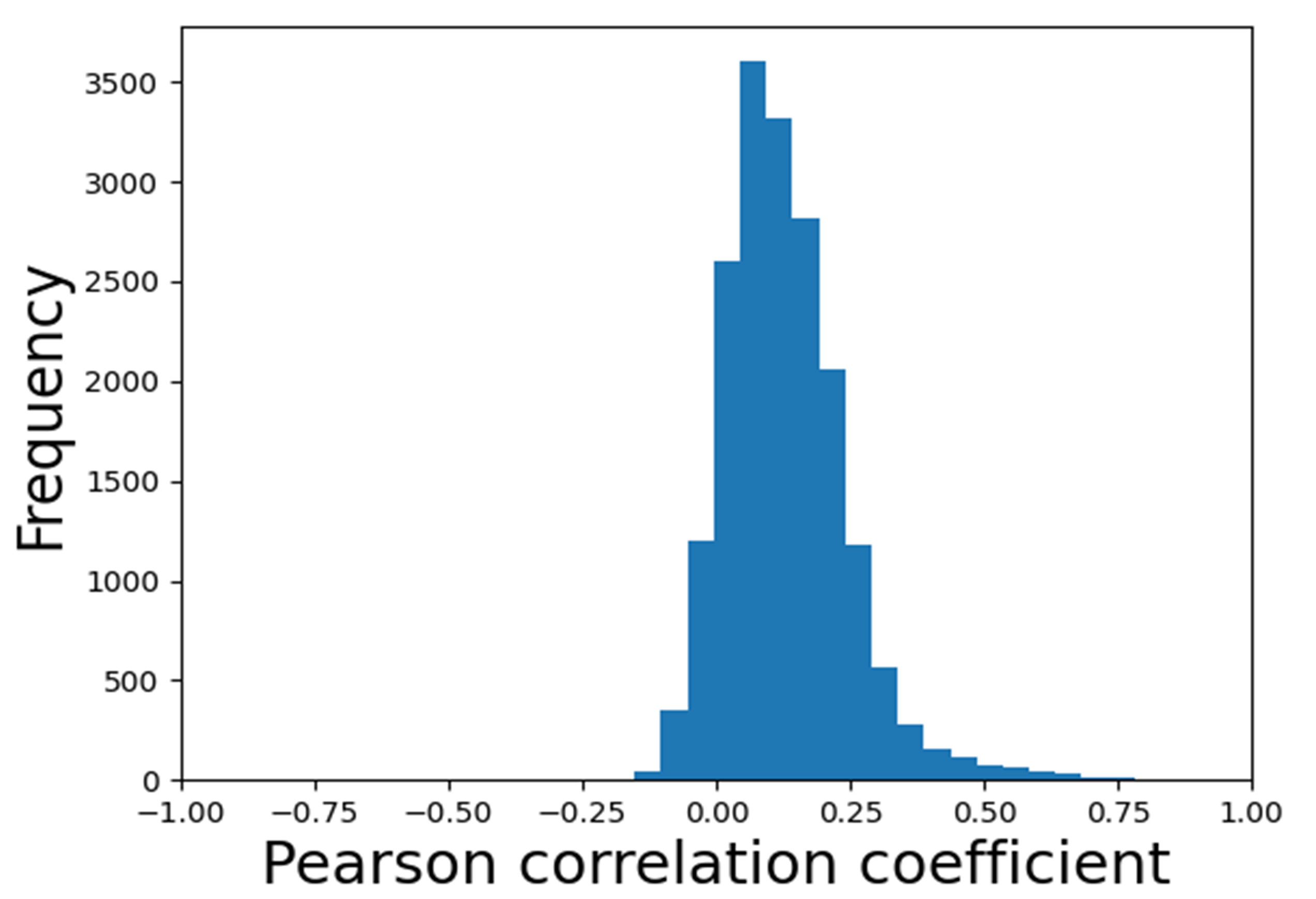


# **Supplementary Figure S5** Histogram of Pearson’s correlation coefficients for all genes. The histogram shows Pearson’s correlation coefficients of 18,542 genes in section C.


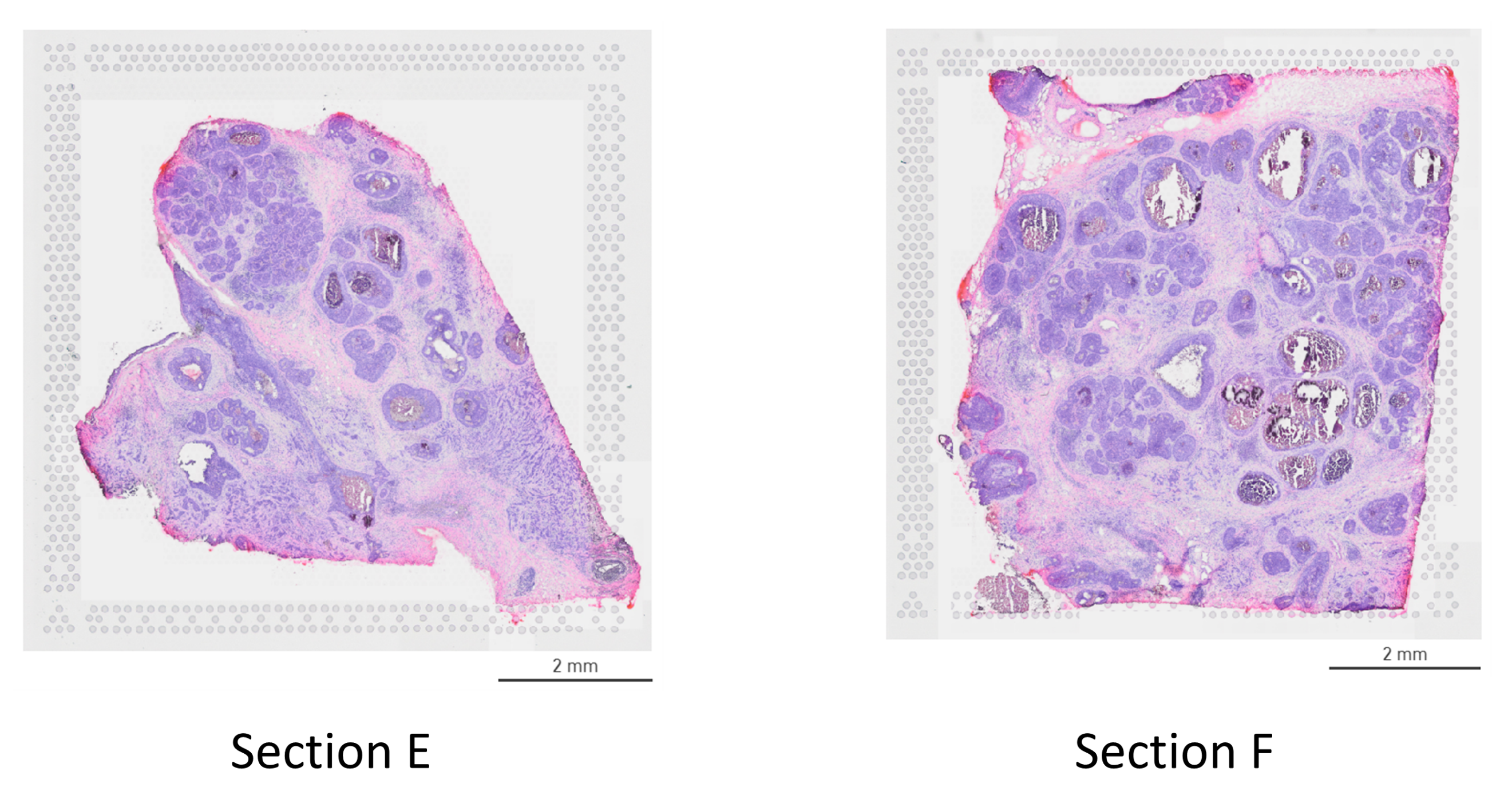


# **Supplementary Figure S6** Human breast cancer tissue sections (sections E and F). Two slide images show H&E-stained human breast cancer tissues from sections E and F. Sections E and F were derived from one patient.

**
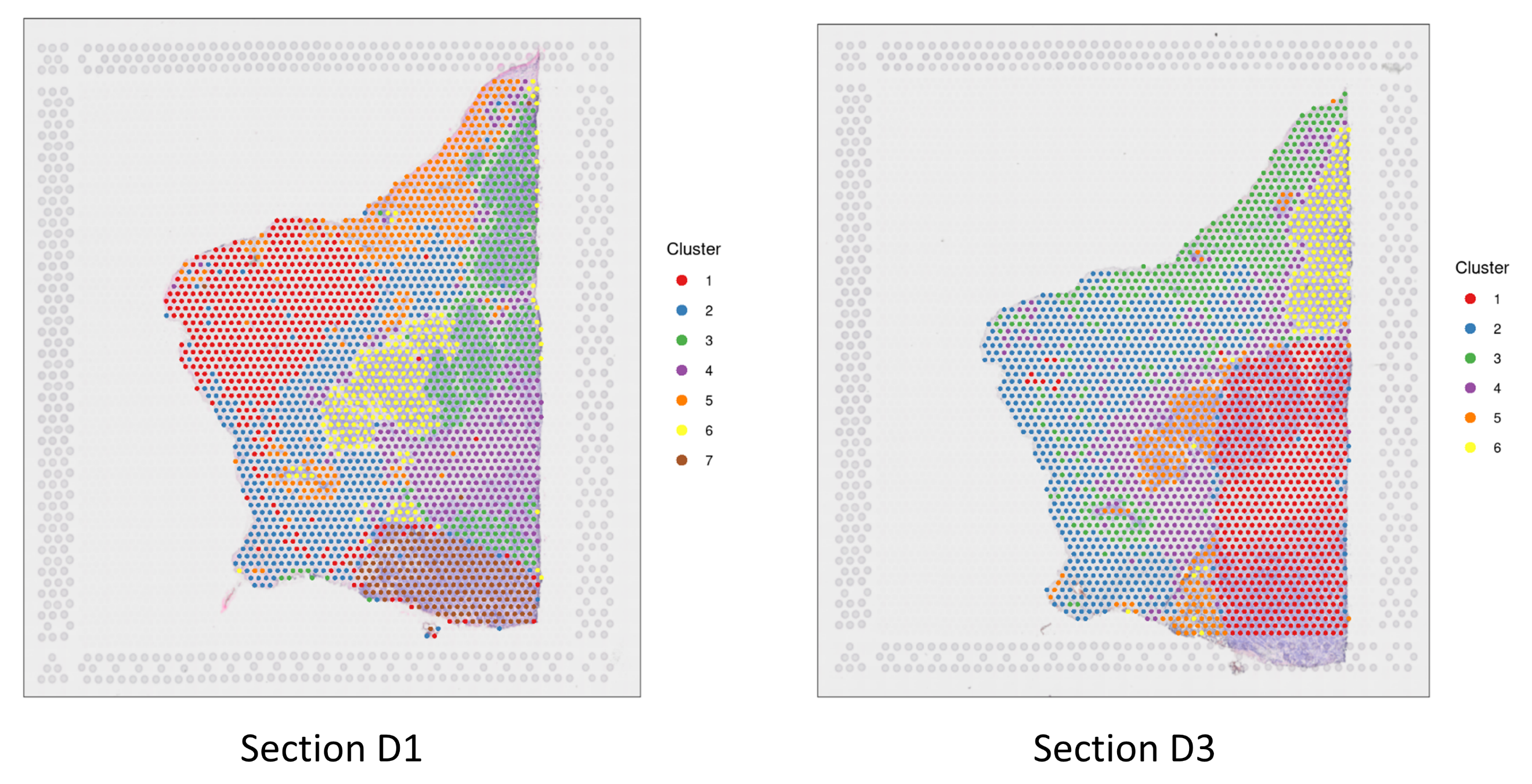
**

# **Supplementary Figure S7** Transcriptomic cluster types. Image on the left shows the cluster types obtained from Space Ranger in section D1. Bottom right of section D1 was classified as cluster 7 because of potential permeabilization errors. Right image shows the cluster types obtained from Space Ranger in section D3. Bottom right of section D3 was classified as cluster 1 because of potential permeabilization errors.


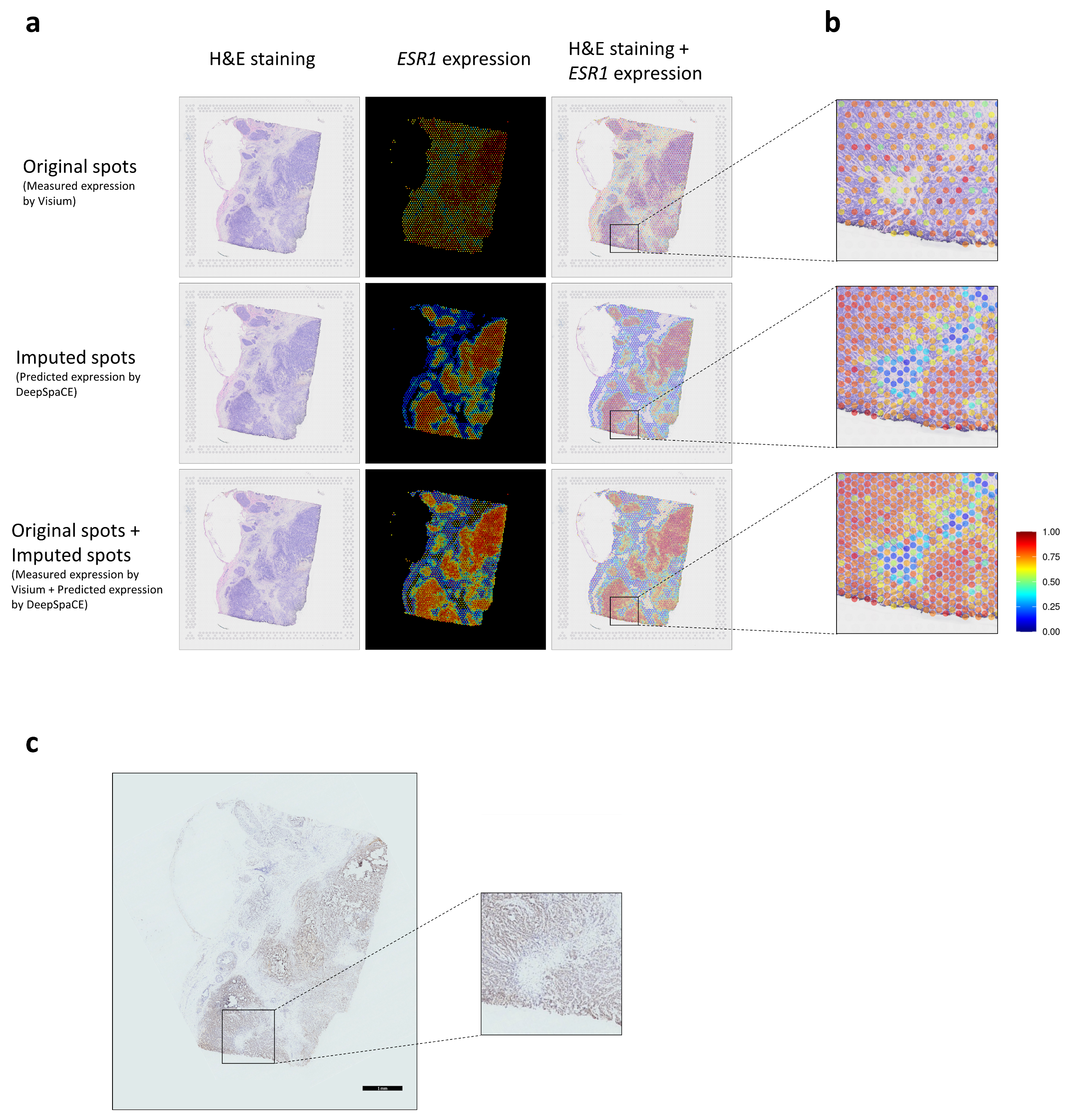


# **Supplementary Figure S8** Super-resolution of *ESR1*. (**a**) Nine images show the super-resolved results of *ESR1* expression. The three images in the left column show section C after H&E staining. The three images on the middle column represent heatmaps of *ESR1* expression in the original spots measured by Visium (top), imputed spots predicted by DeepSpaCE (middle), and both original spots measured by Visium and imputed spots predicted by DeepSpaCE (bottom). The three images on the right columns show *ESR1* expression overlaid on section C after H&E staining. (**b**) Three enlarged images show the region of low *ESR1* expressions. Spot size is adjusted to smaller than the exact spot size of the Visium platform to show the background image. (**c**) Image of section C after immunohistochemical staining of the *ESR1* protein. Right enlarged image is the same region as **Supplementary Fig. S8b**.

**
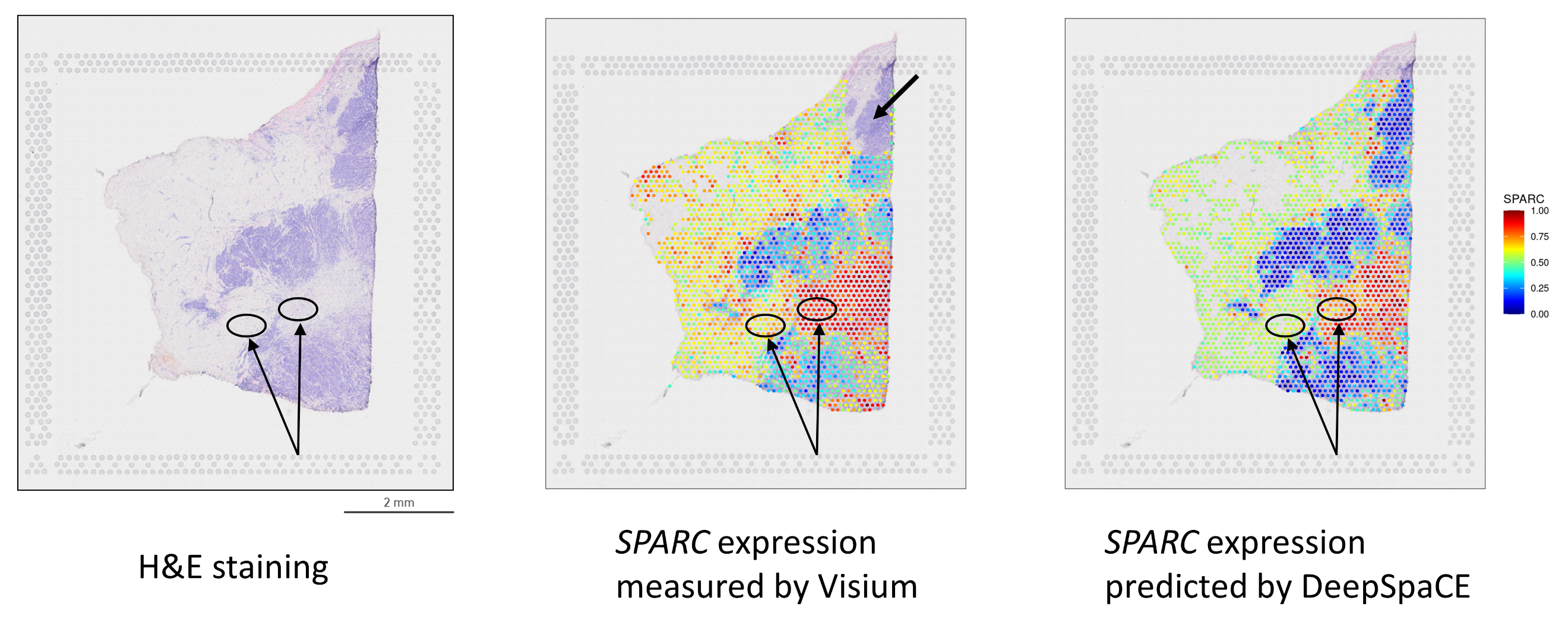
**

# **Supplementary Figure S9** Measured and predicted expression of *SPARC*. Left image shows section D2 after hematoxylin and eosin (H&E) staining. Middle image shows a heatmap of normalized *SPARC* expression in section D2, measured using Visium. *SPARC* expression in the upper right region (black arrow) of section D2 could not be measured, because of potential permeabilization errors. Right image shows the heatmap of *SPARC* expression in section D2, predicted by DeepSpaCE. Blank areas represent spots that were excluded because of a low amount of information.


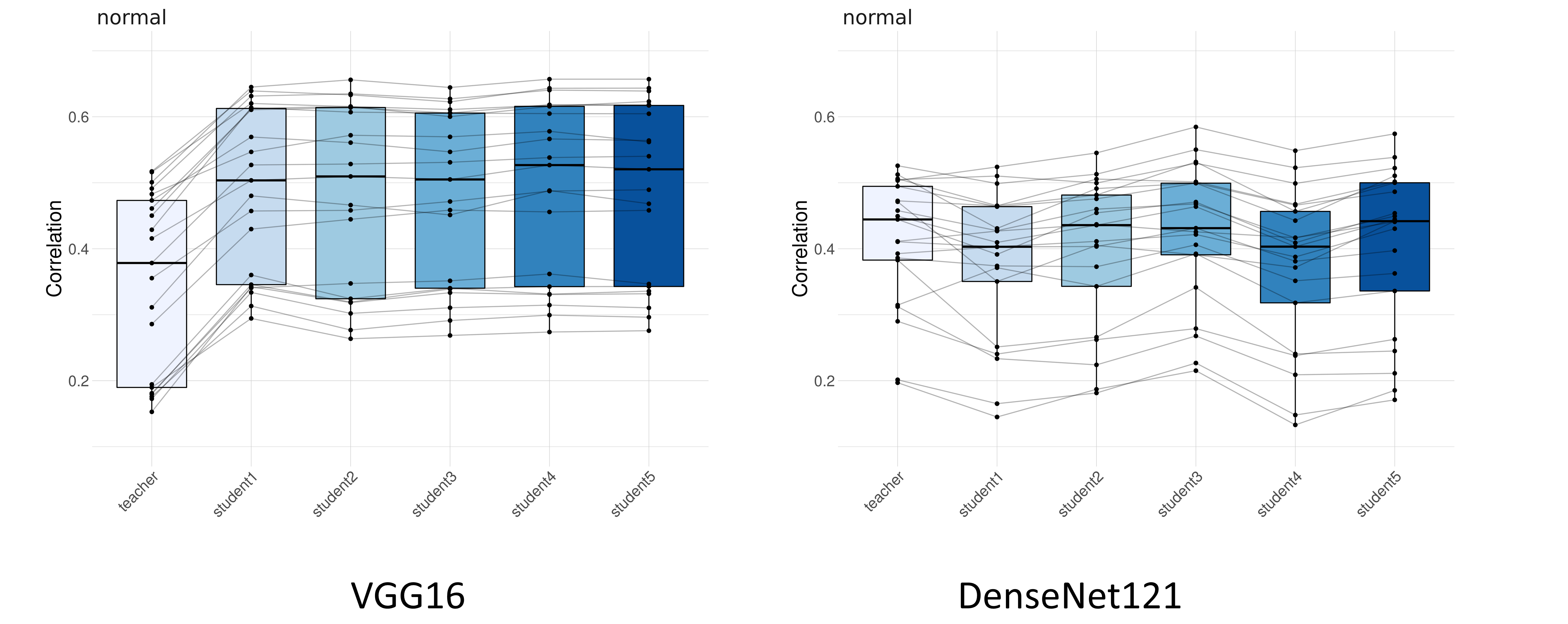


# **Supplementary Figure S10** Semi-supervised learning comparing between VGG16 and DenseNet121. Box plots show Pearson’s correlation coefficients between the measured and predicted gene-expression levels of 21 breast cancer-related microenvironment markers. Left box plot displays the results of VGG16. Right box plots show the semi-supervised learning results of DenseNet121. For the box plot, the box indicates the first and third quartiles; horizontal center line marks the medians; upper whisker extends from the hinge to the highest value that is within 1.5 × interquartile range (IQR) of the hinge; lower whisker extends from the hinge to the lowest value within 1.5 × IQR of the hinge; and data were plotted as points. Black lines between boxes connect the same gene.


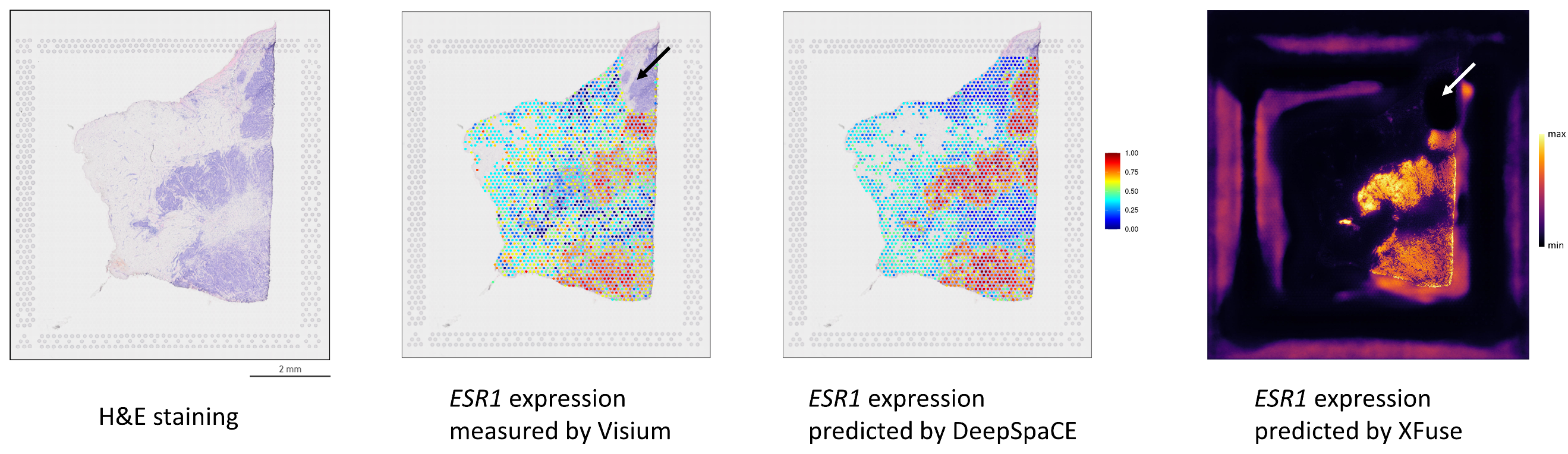


# **Supplementary Figure S11** Comparison between DeepSpaCE and XFuse. The figures show the H&E image, original expression, predicted expression by DeepSpaCE, and the result of XFuse. The black and white arrows show the potential permeabilization error region. The three images from the left are same as **Fig. 2a**.


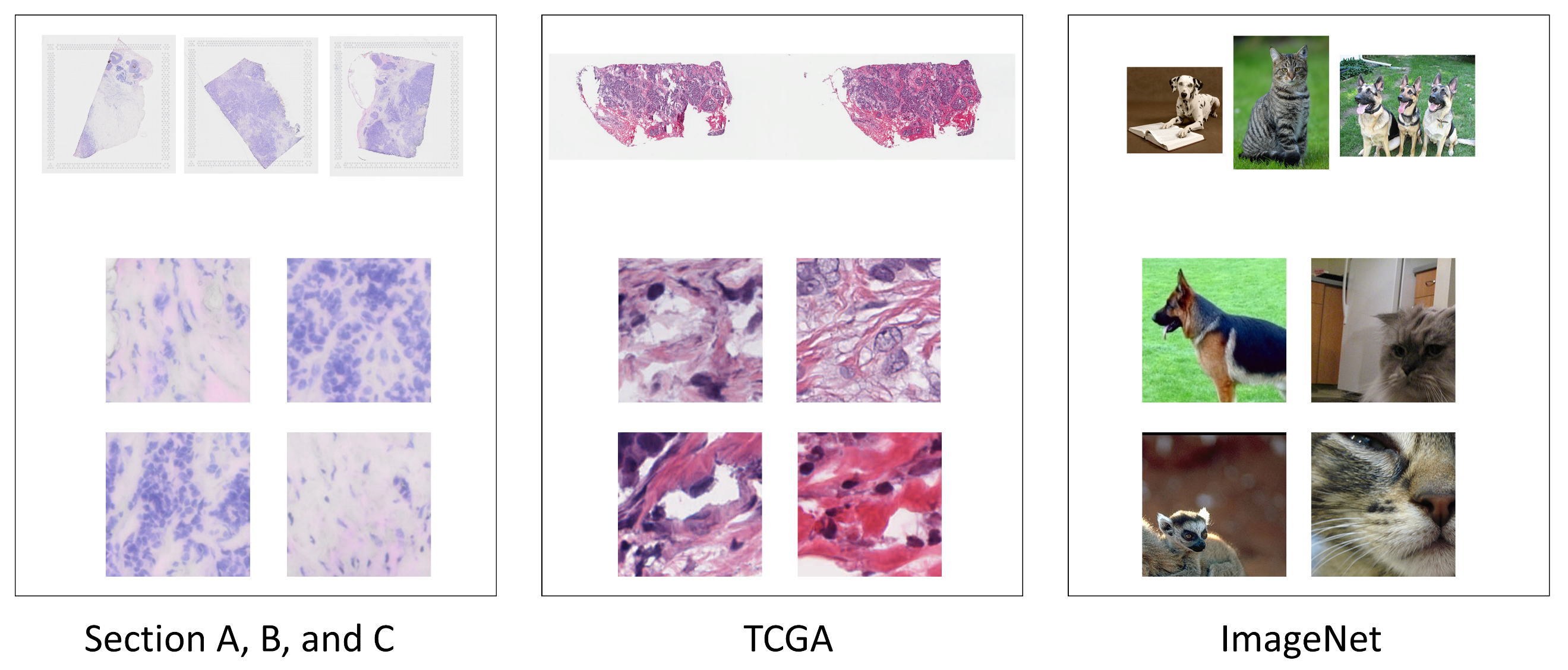


# **Supplementary Figure S12** Examples of unlabeled images used for semi-supervised learning. Left images are derived from sections A–C, center images are obtained from TCGA, and right images are obtained from ImageNet. Original images obtained from TCGA and ImageNet were cropped to 224 × 224 pixels.

**
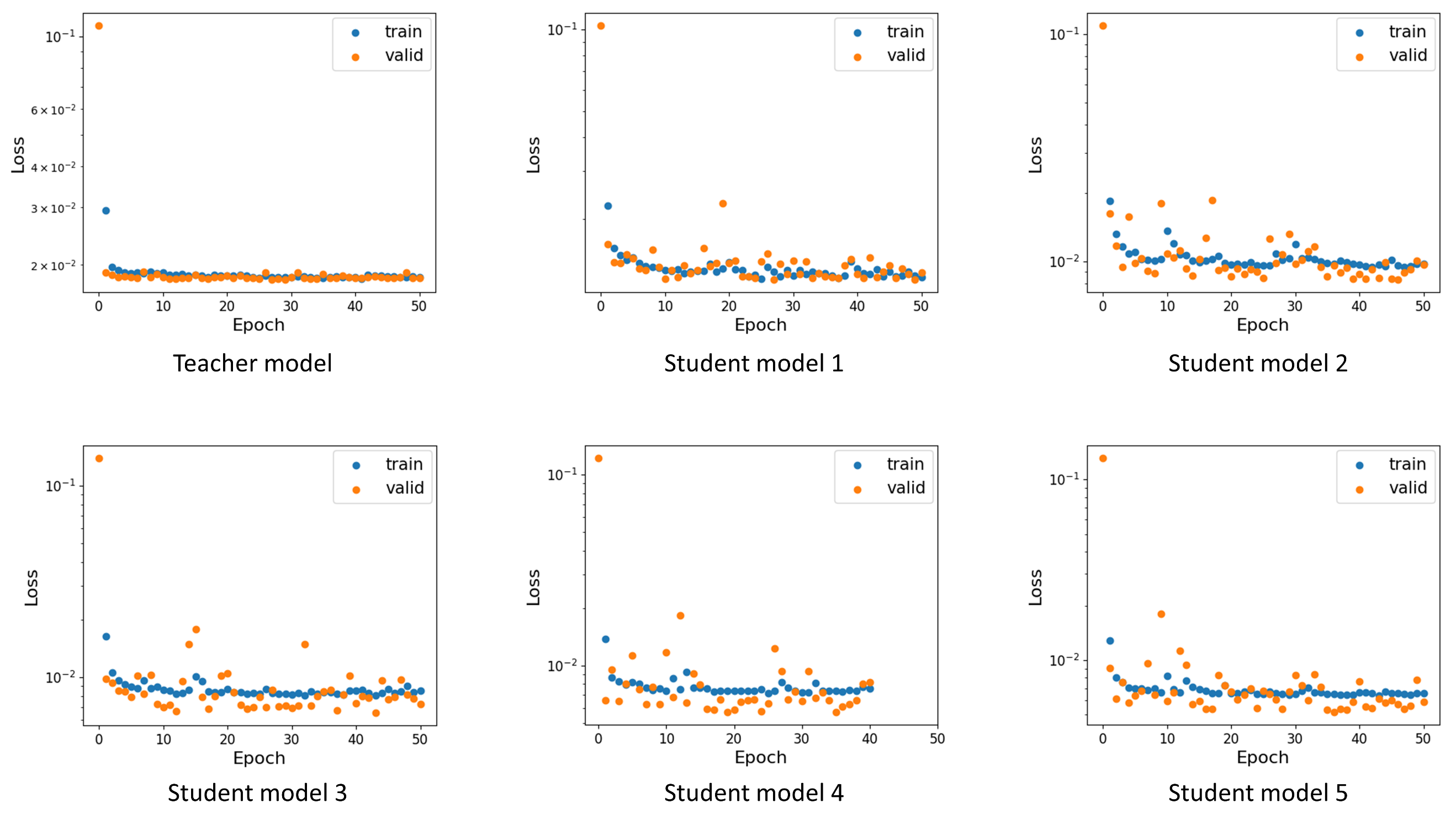
**

# **Supplementary Figure S13** Examples of a learning curve. Scatter plots show the loss values for the training (blue) and validation (orange) datasets. The learning curves were obtained in the semi-supervised learning of 21 breast cancer-related microenvironment marker genes. The training cycle was repeated until 50 epochs. In the case of student model 4, the learning was terminated by early stopping.


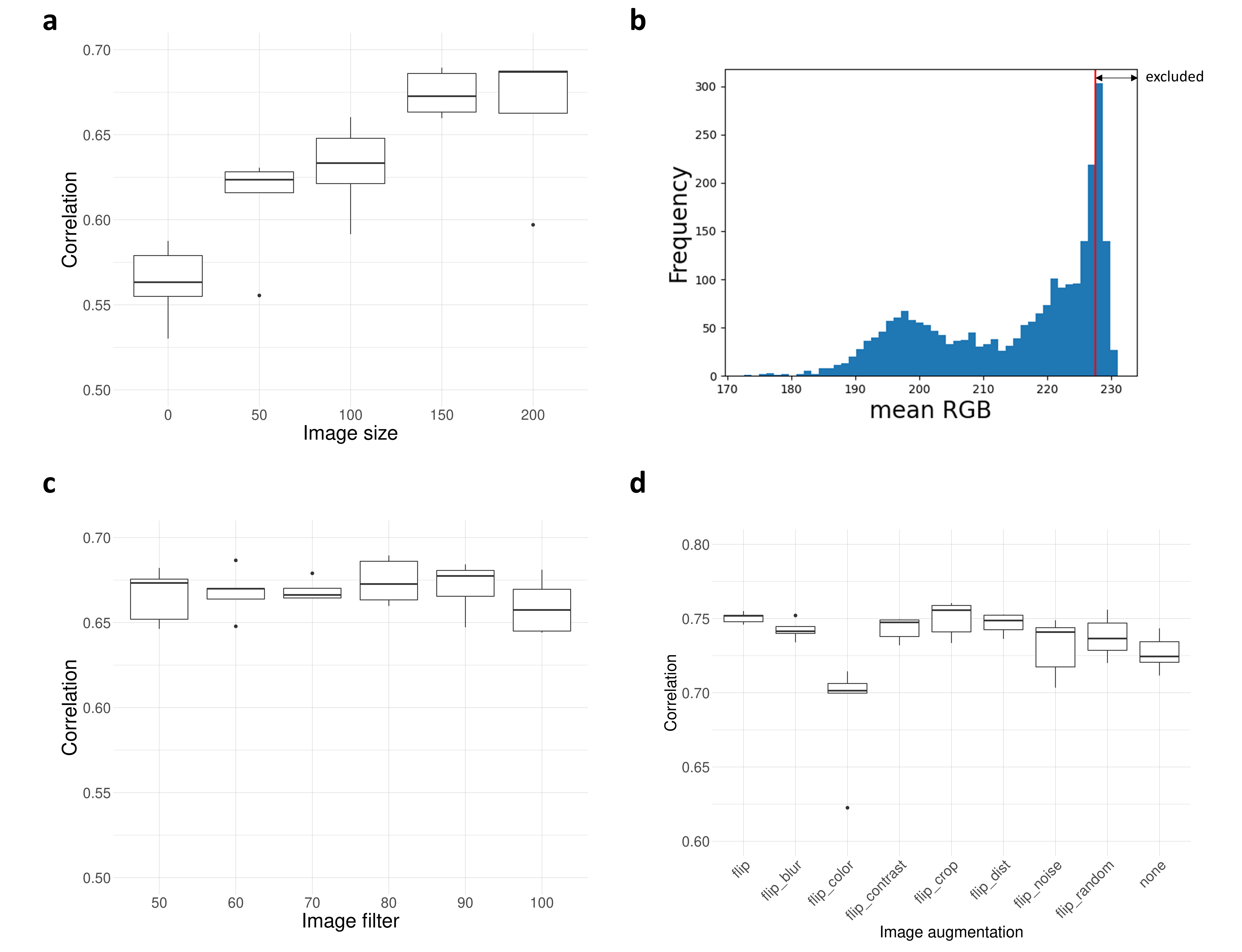


# **Supplementary Figure S14** Parameter optimization of DeepSpaCE. (**a**) Box plot shows the Pearson’s correlation coefficients for each image size (0%, 50%, 100%, 150%, and 200%; relative to the original Visium spot size). (**b**) Histogram shows the mean RGB values in section D2. Spots on the right of the red line were filtered out. (**c**) Box plot shows Pearson’s correlation coefficients for each image-filtering threshold (50%, 60%, 70%, 80%, 90%, and 100%; percentiles of mean RGB values). (**d**) Box plot shows the Pearson’s correlation coefficients for each image-augmentation method (flipping, flipping + blurring, flipping + color, flipping + contrast, flipping + cropping, flipping + distortion, flipping + noise, flipping + random (blurring, distortion, or noise), and none). For the box plot, the box indicates the first and third quartiles; the horizontal center line marks the medians; the upper whisker extends from the hinge to the highest value that is within 1.5 × interquartile range (IQR) of the hinge; the lower whisker extends from the hinge to the lowest value within 1.5 × IQR of the hinge.


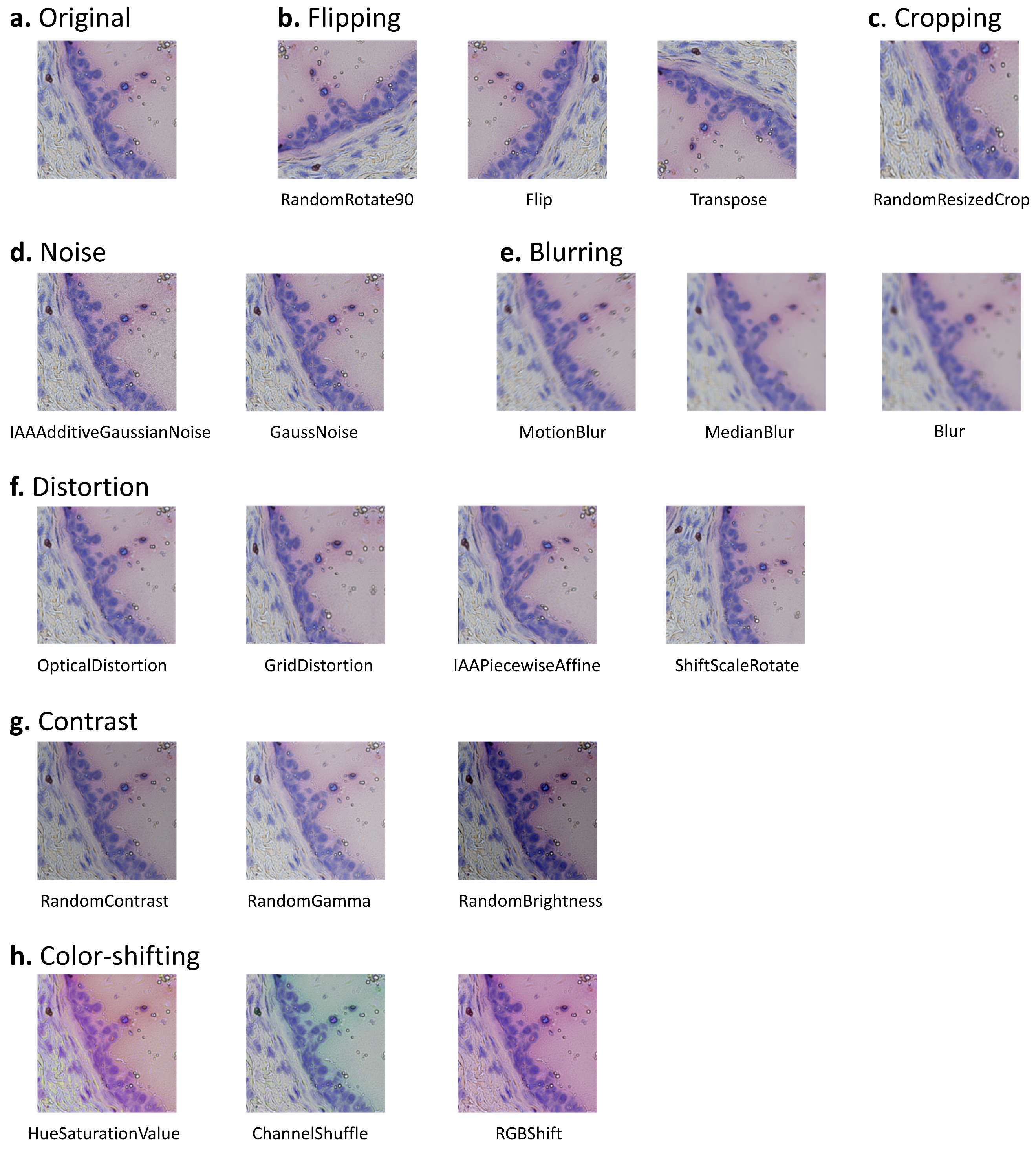


# **Supplementary Figure S15** Examples of image augmentation. Examples of each image augmentation (generated using the Albumentations library) are shown. (**a**) Original image. (**b**) Flipping (RandomRotate90, Flip, and Transpose). (**c**) Cropping (RandomResizedCrop). (**d**) Noise (IAAAdditiveGaussianNoise and GaussNoise). (**e**) Blurring (MotionBlur, MedianBlur, and Blur). (**f**) Distortion (OpticalDistortion, GridDistortion, IAAPiecewiseAffine, and ShiftScaleRotate). (**g**) Contrast (RandomContrast, RandomGamma, and RandomBrightness). (**h**) Color-shifting (HueSaturationValue, ChannelShuffle, and RGBShift).

**
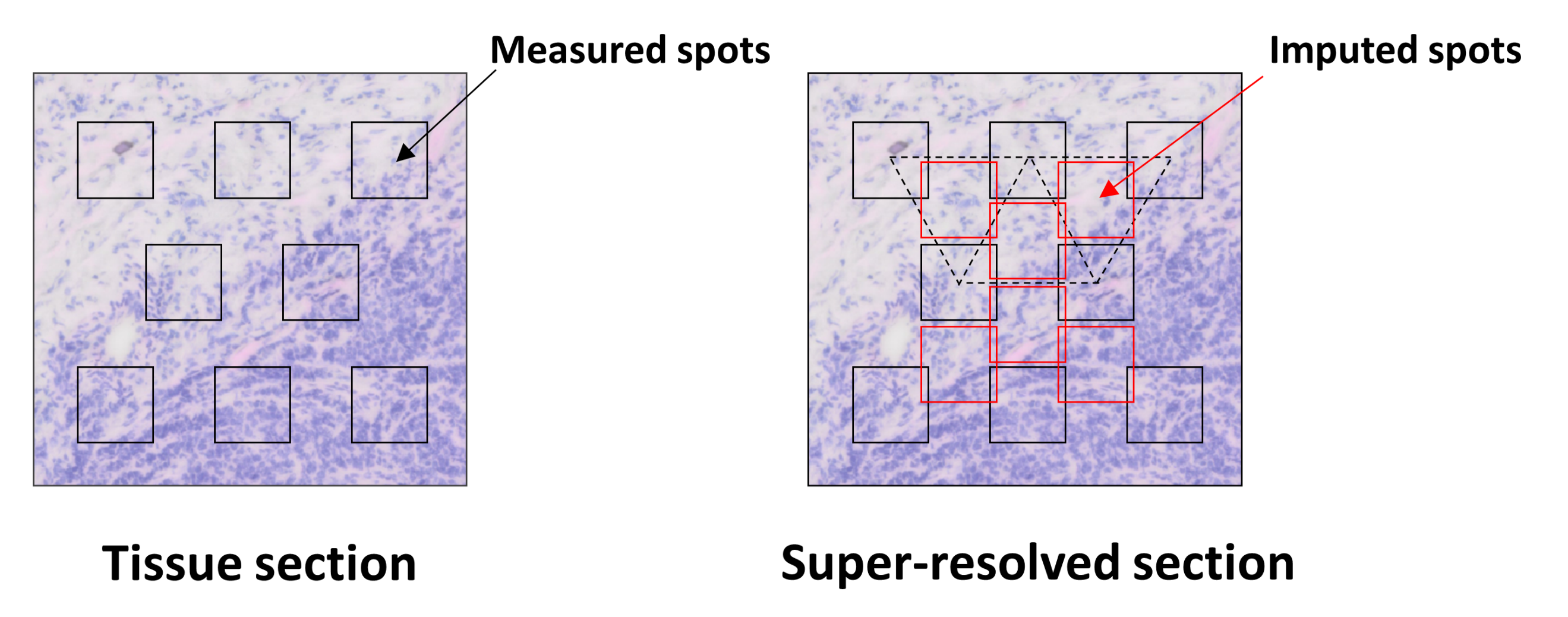
**

# **Supplementary Figure S16** Cropping for super-resolution of section images. Left section image shows the original spots (black) measured by the *in situ* capturing platform. Right section image shows the original spots (black) and imputed spots (red) which is located on the center around three original spots.

**
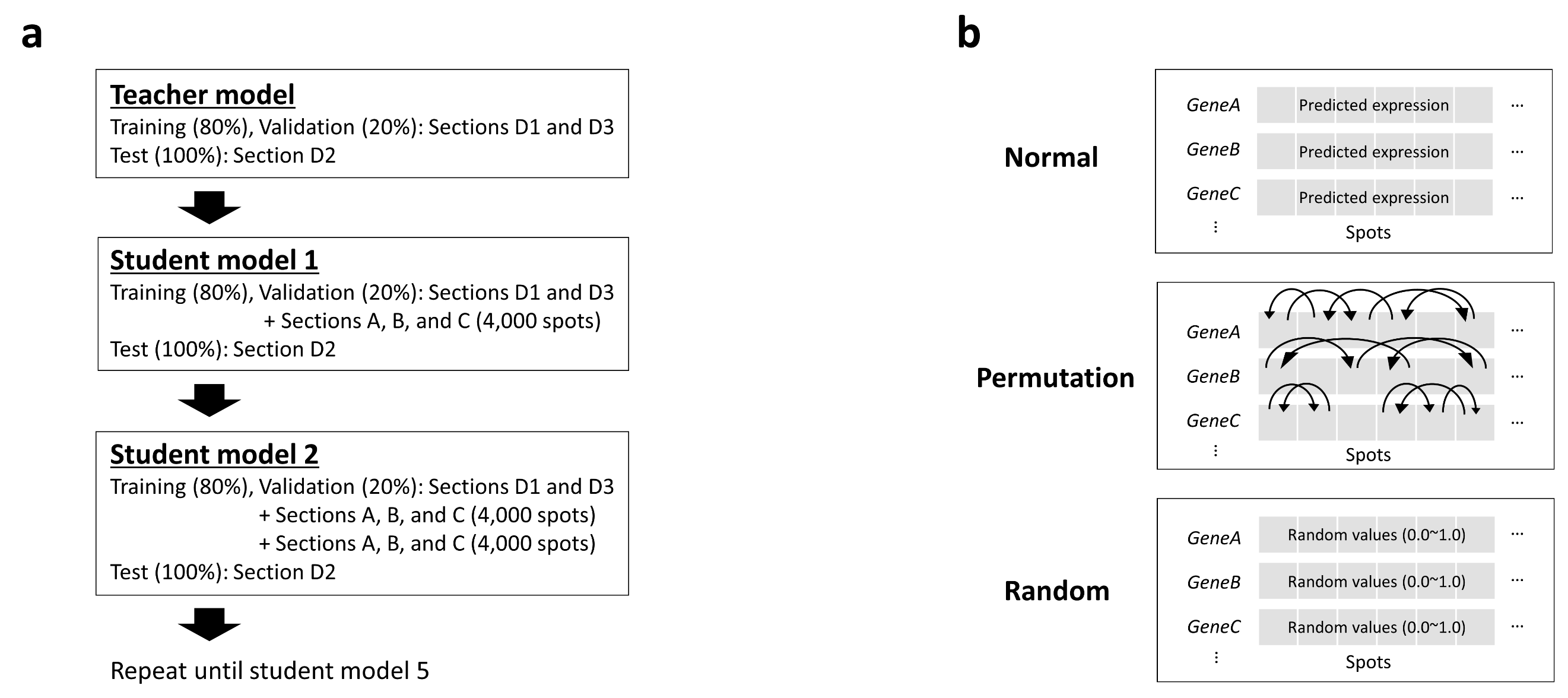
**

# **Supplementary Figure S17** Schematic of semi-supervised learning. (**a**) Schematic of semi-supervised learning showing the dataset for each model in the section imputation. (**b**) We compared three types of gene-expression profiles using semi-supervised methods. In the case of usual values, the predicted expression levels were directly used for semi-supervised learning. In case of permutated values, the predicted expression levels were randomly permutated and used for semi-supervised learning. Random values of zero to one were used for semi-supervised learning.

# **Supplementary Table S1** Prediction accuracy in sections D2. The table shows mean and SD values of Pearson’s correlation coefficients between the measured and predicted expression levels of three breast cancer-marker genes and 21 breast cancer-related microenvironment marker genes. The 5-fold cross-validations were performed using sections D2 as training and test data

# **Supplementary Table S2** Comparison of prediction accuracy between single-task and multi-task leaning. The table shows mean and SD values of Pearson’s correlation coefficients between the measured and predicted expression levels of three breast cancer-marker genes and 21 breast cancer-related microenvironment marker genes. The 5-fold cross-validations of single-task and multi-task learning were performed using section C as training and test data.

# **Supplementary Table S3** Gene set enrichment analysis (GSEA) in section C. GSEA was performed to characterize highly predictable genes using 18,451 genes expressed in section C. The table shows the result of GSEA (FDR q-value<0.05); NAME (Gene set name), SIZE (Number of genes in the gene set after filtering out those genes not in the expression dataset), ES (Enrichment score for the gene set), NES (Normalized enrichment score), NOM p-value (Nominal p value), FDR q-value (False discovery rate), FWER p-value (Familywise-error rate), and RANK AT MAX (The position in the ranked list at which the maximum enrichment score occurred).

# **Supplementary Table S4** Prediction accuracy in sections E and F. The table shows mean and SD values of Pearson’s correlation coefficients between the measured and predicted expression levels of three breast cancer-marker genes and 21 breast cancer-related microenvironment marker genes. The 5-fold cross-validations were performed using sections C as training data and section E and F as test data.

# **Supplementary Table S5** Summary statistics of cluster-type prediction. The table shows micro and macro precision, recall, f1-score, AUC, 95% CI of AUC, and number of samples.

# **Supplementary Table S6** Top5 pathways of cluster 3 in section D1, cluster 3 in section D2, and cluster 6 in section D3. The table shows the result top5 pathways of cluster 3 in section D1, cluster 3 in section D2, and cluster 6 in section D3 (adjusted P-value < 0.05 (Benjamini-Hochberg method)).

# **Supplementary Table S7** Top5 pathways in section D2. The table shows top5 pathway analysis in section D2 (adjusted P-value < 0.05 (Benjamini-Hochberg method)).

# **Supplementary Table S8** Semi-supervised learning (Training: sections D1 and D3, Test: section D2). The table shows mean and SD values of Pearson’s correlation coefficients between the measured and predicted expression levels of 21 breast cancer-related microenvironment marker genes. The 5-fold cross-validation of semi-supervised learning was performed using sections D1 and D3 as training data, section D2 as test data, and sections A–C as unlabeled data.

# **Supplementary Table S9** Semi-supervised learning (Training: sections D2 and D3, Test: section D1). The table shows mean and SD values of Pearson’s correlation coefficients between the measured and predicted expression levels of 21 breast cancer-related microenvironment marker genes. The 5-fold cross-validation of semi-supervised learning was performed using sections D2 and D3 as training data, section D1 as test data, and sections A–C as unlabeled data.

# **Supplementary Table S10** Semi-supervised learning (Training: sections D1 and D2, Test: section D3). The table shows mean and SD values of Pearson’s correlation coefficients between the measured and predicted expression levels of 21 breast cancer-related microenvironment marker genes. The 5-fold cross-validation of semi-supervised learning was performed using sections D1 and D2 as training data, section D3 as test data, and sections A–C as unlabeled data.

# **Supplementary Table S11** Comparison of prediction accuracy between VGG16 and DenseNet121. The table shows mean and SD values of Pearson’s correlation coefficients between the measured and predicted expression levels of 21 breast cancer-related microenvironment marker genes. The 5-fold cross-validation of semi-supervised learning was performed using sections D1 and D3 as training data, section D2 as test data, and sections A–C as unlabeled data.

# **Supplementary Table S12** Semi-supervised learning of cluster-type prediction. The table shows mean and SD values of accuracy in teacher and student 1–5 models.

# **Supplementary Table S13** Tissue section information. The table shows the original tissue section image size, spot image size, and number of spots measured with Visium platform in sections A–C and D1–D3.

# **Supplementary Table S14** Number of samples in training, validation, and test. The table shows the number of spots for training, validation, and testing.

# **Supplementary Table S15** Pathway analysis of each cluster in section D2. The table shows all pathways of each cluster in section D2.
